# Use of a health worker-targeted smartphone app to support quality malaria RDT implementation in Busia County, Kenya: A feasibility and acceptability study

**DOI:** 10.1371/journal.pone.0295049

**Published:** 2024-03-26

**Authors:** Malia Skjefte, Shawna Cooper, Stephen Poyer, Christopher Lourenço, Sam Smedinghoff, Brett Keller, Tonny Wambua, Christine Oduor, Sasha Frade, Wycliffe Waweru

**Affiliations:** 1 Malaria Department, Population Services International (PSI), Washington, DC, United States of America; 2 Audere, Seattle, WA, United States of America; 3 Digital Health & Monitoring Department, Population Services International (PSI), Nairobi, Kenya; Menzies School of Health Research, AUSTRALIA

## Abstract

Malaria rapid diagnostic tests (mRDTs) are an essential diagnostic tool in low-resource settings; however, administration and interpretation errors reduce their effectiveness. *HealthPulse*, a smartphone mRDT reader application, was developed by Audere to aid health workers in mRDT administration and interpretation, with an aim to improve the mRDT testing process and facilitate timely decision making through access to digitized results. Audere partnered with PSI and PS Kenya to conduct a pilot study in Busia County, Kenya between March and September 2021 to assess the feasibility and acceptability of *HealthPulse* to support malaria parasitological diagnosis by community health volunteers (CHVs) and private clinic health workers (private clinic HWs). Metadata was interpreted to assess adherence to correct use protocols and health worker perceptions of the app. Changes to mRDT implementation knowledge were measured through baseline and endline surveys. The baseline survey identified clear mRDT implementation gaps, such as few health workers correctly knowing the number of diluent drops and minimum and maximum wait times for mRDT interpretation, although health worker knowledge improved after using the app. Endline survey results showed that 99.6% of health workers found the app useful and 90.1% found the app easy to use. Process control data showed that most mRDTs (89.2%) were photographed within the recommended 30-minute time frame and that 91.4% of uploaded photos passed the app filter quality check on the first submission. During 154 encounters (3.5% of all encounters) a health worker dispensed an artemisinin-based combination therapy (ACT) to their patient even with a negative mRDT readout. Overall, study results indicated that *HealthPulse* holds potential as a mobile tool for use in low-resource settings, with future supportive supervision, diagnostic, and surveillance benefits. Follow-up studies will aim to more deeply understand the utility and acceptance of the *HealthPulse* app.

## 1. Introduction

Nearly half of the world’s population is at risk of malaria, a vector-borne parasitic disease transmitted through the bite of an infected *Anopheles* mosquito [1,2]. The World Health Organization (WHO) African Region alone accounted for roughly 95% of malaria cases and 96% of malaria deaths, globally, in 2021. Overall, Kenya has made great progress in reducing the burden of disease over the last decade [3]. Between 2010 and 2020, malaria prevalence dropped by 50% (38.1% to 18.9%) in the county’s lake-endemic region (in western Kenya) and by 49% (11.4% to 5.8%) nationwide [4]. However, malaria remains a significant public health concern as approximately 75% of Kenya’s population is at risk of infection, with an increased burden in the country’s western regions [5].

The WHO guidelines for malaria case management recommend diagnosing all suspected malaria cases with a parasitological test (microscopy or rapid diagnostic test (RDT)) and treating confirmed, uncomplicated malaria cases with an artemisinin-based combination therapy (ACT) [6]. Following the guidelines, in 2012, the Government of Kenya (GoK) launched the use of malaria rapid diagnostic test (mRDT) kits with the goal of ensuring effective and timely malaria case management and promoting stronger surveillance systems [7]. The Kenya Malaria Strategy (KMS) (2019–2023) set an associated objective of managing 100% of suspected malaria cases according to national treatment guidelines, which require parasitological confirmation of every case [8]. Additionally, the strategy focuses on making malaria treatment accessible by supporting community case management of malaria (CCMm) using community health volunteers (CHVs) [9].

In Kenya, mRDTs are used by a variety of healthcare workers, including private clinic health workers (private clinic HWs) and CHVs trained to provide health prevention, promotion, and community referral services [9]. mRDTs have demonstrated reliability in countries such as Kenya where there is limited access to microscopy, especially in low-resource settings where laboratories are not easily accessible [10,11]. Therefore, it is crucial to ensure that health workers can correctly administer mRDTs and interpret the test results, followed by the provision of appropriate treatment for confirmed malaria cases. Broader studies show that using mRDTs for parasitological diagnosis can improve health outcomes and reduce the cost of malaria care in endemic regions throughout sub-Saharan Africa [12–14]. Multiple studies have also demonstrated the cost-effectiveness of mRDTs compared to microscopy or presumptive treatment, showing potential for use in low-resource settings [15–17]. However, these gains can be thwarted when mRDTs are misadministered or misinterpreted by health workers [18,19].

Multiple mobile health (mHealth) interventions have been developed in recent years to support health workers in mRDT administration and interpretation. For example, the Deki Reader™, a point of care testing (POCT) platform developed by Fio Corporation, provides automated RDT analysis and interpretation for a variety of diseases (including malaria, HIV, and Ebola virus) by using image analysis software and automated data generation [20]. While studies have identified potential for the Deki Reader™ as a useful malaria diagnosis, treatment, and data management tool, researchers and health workers have highlighted many limitations in using this reader in the field. These limitations include its low battery life, low functionality in high-dirt environments, and difficulty to use among unskilled health workers [21,22]. Similar results were found in a comparative evaluation of another mHealth tool used for reading and interpreting mRDTS [23]. Other POCT reader devices include handheld lateral flow immunoassays (LFIA) [24,25] and the mHAT RDT reader application (app) [26], although the functionality of these devices in the field is still limited and being explored [27].

Expanded access to mobile phones has led to the development of an array of mHealth interventions to aid timely and accessible POCT, although challenges still exist with their adoption and integration into everyday use by health workers. In Kenya, mobile phones are used by the majority of individuals, with 26.5 million smartphones connected to a network in March 2022, indicating that approximately 54.6% of the population has access to smartphone technology [28,29]. Accessibility of phones among Kenyans, paired with high public health needs, makes it feasible to accelerate the uptake of mHealth interventions in the country, although key barriers to adoption and integration into the health system still exist [30]. While a systematic review of mHealth interventions in Kenya found that current mHealth interventions were effective in collecting and transferring patient data and providing remote care management (including diagnosis, treatment, and follow-up), low will among health workers to use the intervention among an already resource-strained health system may limit the potential to scale up and integrate some of these interventions [30]. Feasibility and health worker acceptability of new mHealth interventions must therefore be considered and further explored when new interventions are developed.

With a goal of optimizing accessible mHealth interventions in resource constrained environments, the Seattle-based global digital health nonprofit Audere, developed *HealthPulse*, a mobile mRDT reader app that unifies mRDT administration guidance, patient data digitization, mRDT photo capture, interpretation guidance, and tracking of antimalarial dispensing practices [31]. The app does not provide an interpretation of the mRDT to the health worker, but rather guides them through proper mRDT use, photo capture, and interpretation. The timely data collected through HealthPulse positions the app as a supervision, surveillance, and stock management tool, apart from its key function to improve the mRDT testing process and outcomes for health workers.

In partnership with two global health nonprofits, Population Services International (PSI) and Population Services Kenya (PS Kenya), Audere implemented a pilot study to evaluate the feasibility, acceptability, and effectiveness of the app in community clinics and the private sector. While a study evaluating the effectiveness of the app is presented elsewhere, this paper aims to understand the feasibility of *HealthPulse* implementation among health workers in a low-resource setting. Additionally, this paper focuses on analyzing the acceptability of the app among health workers, investigating whether the app can be easily integrated into health workers’ standard workflow and understanding how it was perceived as a mRDT testing support tool. Results from this study will be used to guide additional development and utilization of the *HealthPulse* app throughout Kenya and other high burden geographies.

## 2. Materials and methods

### 2.1 Study area

**Fig 1:**
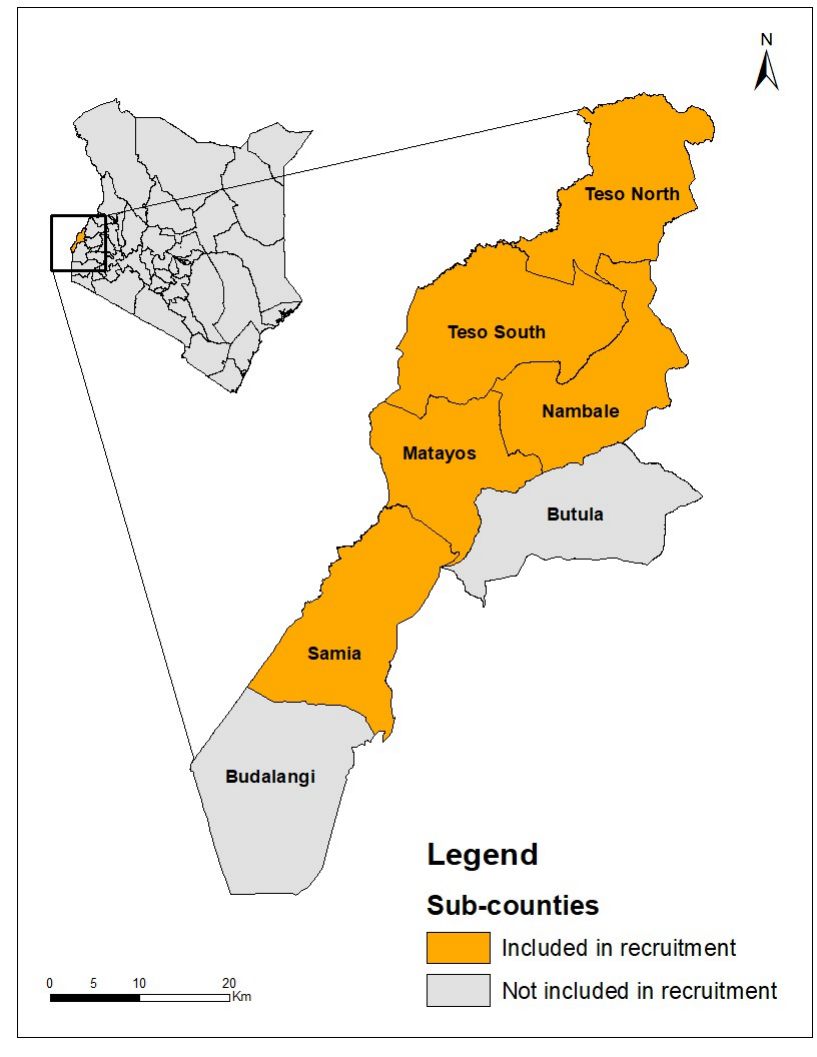
Busia County, Kenya and its seven sub-counties. This map was created using ArcMap 10.8.2, a product of Esri. All map layers were retrieved from The Humanitarian Data Exchange, which include the administrative levels 0–2 from the OCHA Regional Office for Southern and Eastern Africa (ROSEA). A link to the layers is available here: https://data.humdata.org/dataset/cod-ab-ken.

Busia County belongs to Kenya’s lake endemic region, which had the highest prevalence of malaria among children aged 6 to 14 in 2020 according to mRDT (22.8%) and microscopy (18.9%) results [7]. Busia, with an approximate area of 1,700 square kilometers, is bordered to the south by Lake Victoria and comprises seven sub-counties: Budalangi, Samia, Nambale, Butula, Matayos, Teso North, and Teso South. The study site was chosen due to its high malaria endemicity and existing partnership with PS Kenya. The sub-counties Butula and Budalangi were not included in this study to prevent potential confounding as they were already participating in another app-based health intervention. Fig 1 presents the location of Busia County within Kenya and its respective sub-counties.

### 2.2 Study design

**Fig 2:**
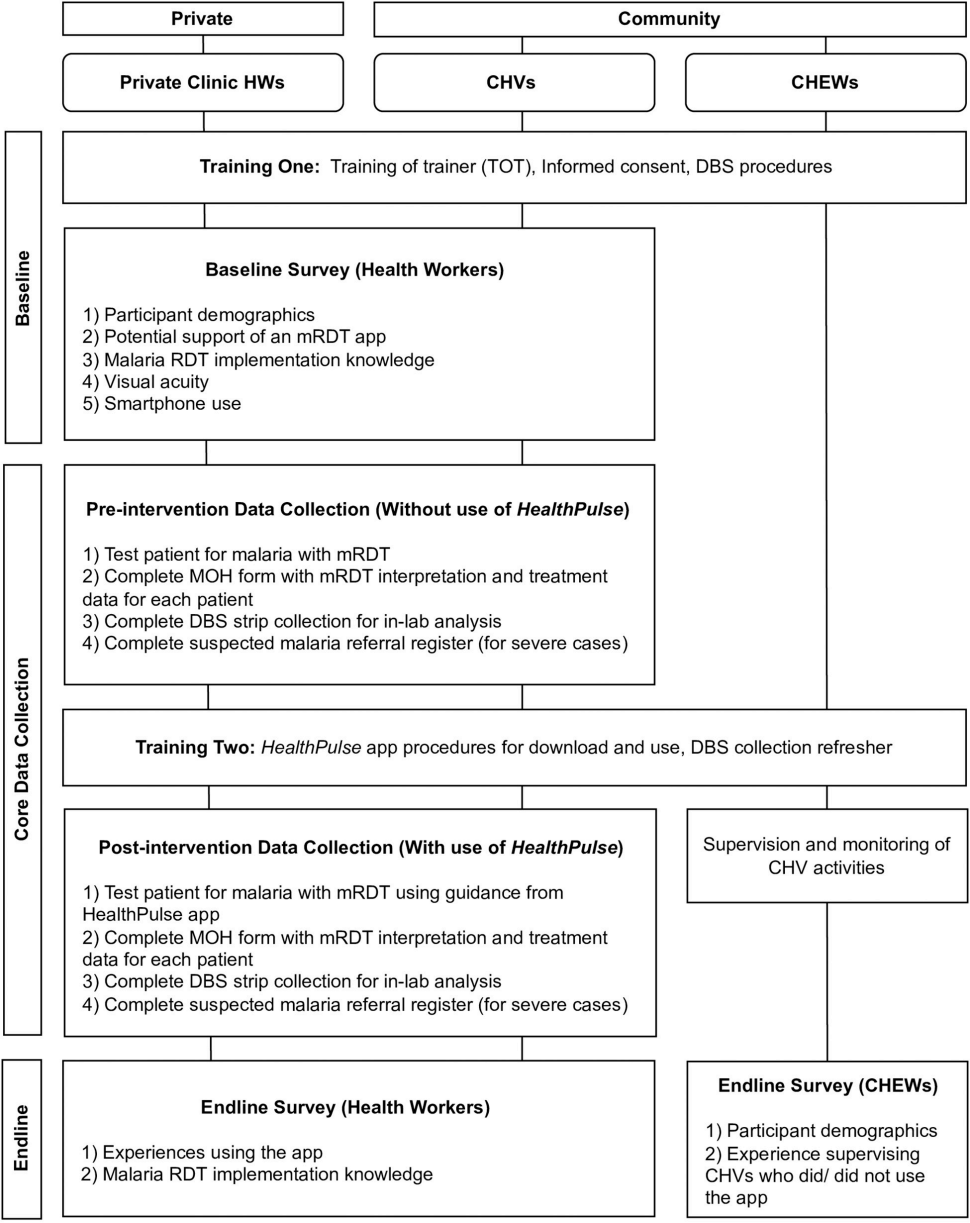
Study design and workflow.

Quantitative data for this study were collected between March to September 2021. The study used a pre- and post-quantitative design to compare the accuracy of health worker mRDT interpretations before and after the *HealthPulse* app was introduced, referenced as the pre-intervention and post-intervention phases respectively. Baseline and endline surveys were completed by health workers before and after the app was introduced to gauge acceptability and feasibility of app implementation. Routine process data from the app, including health worker interactions, stock levels, submitted test results and ACT dispensal, were recorded during the post-intervention phase. This paper presents the app acceptability and feasibility results; mRDT accuracy results are presented separately. The full study design and workflow is presented in Fig 2.

### 2.3 Study recruitment

The study population was composed of three groups: 1) CHVs, 2) private clinic HWs, and 3) community health extension workers (CHEWs). CHVs and CHEWs work in the public sector. Each CHEW is associated with a public health facility and supervises up to 10 CHVs. Private clinic HWs are not supervised by CHEWs; they report directly to health facility managers or owners. Unlike CHVs and private clinic HWs, CHEWs were not responsible for administering mRDTs using the *HealthPulse* app; therefore, the study team collected CHEW experiences supervising those participating in the intervention.

Recruitment of health workers took place in the five sub-counties described in Fig 1. All private clinics were recruited directly from an existing PS Kenya malaria case management program, which provided mRDT training to health workers and supplied clinics with testing and treatment items (mRDTs and ACTs) from the Ministry of Health (MOH). In total, 41 private clinics agreed to participate in this study. To recruit CHVs, the study team targeted all the community health units in the five sub-counties which were participating in the PS Kenya malaria case management program. The study team reached out to CHEWs to participate, who then led the recruitment of CHVs in their unit. The PS Kenya team ensured that all health workers were recruited openly, fairly, and without pressure.

No formal sample size was set for the acceptability and feasibility outcomes presented in this paper. Overall, 275 CHVs and 47 private clinic HWs completed training one at study initiation, 225 CHVs and 29 private clinic HWs completed training two to introduce the app, and 203 CHVs and 29 private clinic HWs completed the endline survey. After cleaning the data collected through the app, the team removed three health workers from the analysis who did not have data that was matched to a health worker name from the registration list. Such instances were caused when the health worker accidentally deleted the app or switched phones during the study and registered under a different name. The final sample size for this acceptability and feasibility analysis included 200 CHVs and 23 private clinic HWs.

**Fig 3:**
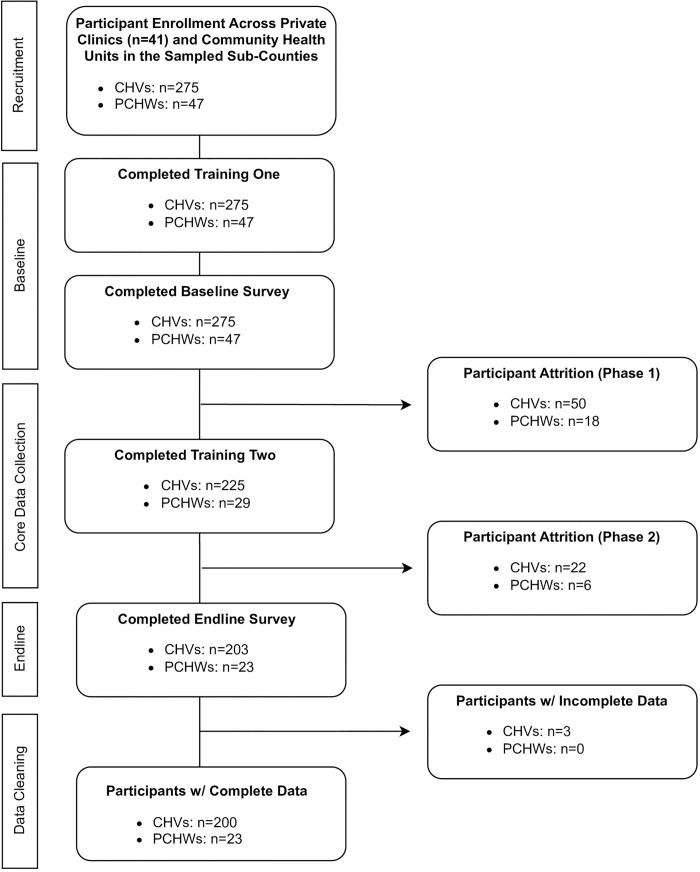
CHV and private clinic HWs sample sizes.

Patients who accessed health services at recruited facilities or via CHVs in their community were informed about the study by their frontline health worker when they presented with suspected malaria symptoms. Forty-five (45) CHEWs opted to participate in the study to supervise CHV *HealthPulse* use during the post-intervention phase and complete an endline survey. Health worker sample sizes for each stage of the study are presented in Fig 3 and additional details on the definition for each group as well as the inclusion and exclusion criteria of the study population are presented in S1 Table.

### 2.4 Staff and health worker training

#### 2.4.1 Training one

The first set of training and baseline survey data collection took place between March 8–20, 2021. A one-day Training of trainers (TOT) was led by a PSI Research Manager to train the county and PS Kenya program staff about the study procedures. Staff included five program research assistants, a county malaria coordinator and county-level MOH staff. The TOT session was followed by a two-day training led by the same PSI Research Manager for all health workers. During this training, health workers were taught how to introduce the study to patients, obtain informed consent, and perform a Dried Blood Spot (DBS) collection, which was used to compare to mRDT accuracy. They also participated in role play and group or paired practical exercises. Health workers did not receive training on mRDT administration and interpretation.

#### 2.4.2 Training two

After the baseline data collection, a second training was held to introduce health workers to the *HealthPulse* app. This one-day training was led by the PSI Research Manager with support from PS Kenya program staff and county malaria coordinators, and held separately for each of the five sub-counties. During training, health workers were guided through the app download process, created an on-device account with a numeric password, received instructions on how to use the app, and received refresher training on the DBS collection process. Most of the training was spent practicing using the app. While health workers were introduced to correct mRDT administration and interpretation protocols in the app, they did not receive specific training on mRDT procedures during this training.

### 2.5 Data collection

#### 2.5.1 Baseline health worker survey

The baseline health worker survey was completed during the first training session. CHVs and private clinic HWs completed a paper survey, which was later transcribed into an online database. This survey collected information on demographics, visual acuity, phone use, pre-intervention attitudes towards using a supportive mRDT reader app, and malaria implementation knowledge. The survey was jointly designed by the study team and the MOH, and was usability tested with a random sample of health workers before deployment.

#### 2.5.2 Pre-intervention core data collection

Core outcome data was collected before and after the *HealthPulse* app was introduced. During the pre-intervention phase, health workers were asked to test patients for malaria and complete two forms, which were an existing part of the health workers’ standard workflow. These included 1) an MOH form with their mRDT interpretation and treatment data for each patient and 2) a suspected severe malaria referral form. During the pre-intervention phase, interpretation and treatment data were collected from the MOH form and analyzed as part of the effectiveness study. No data from the severe malaria referral form was stored or analyzed for the study. Additionally, health workers were required to complete a DBS form for each patient, which was sent for lab analysis by polymerase chain reaction (PCR). Pre-intervention data collection was set to reach completion after 1040 mRDT interpretations were received from CHVs and 1632 mRDT interpretations were received from private clinic HWs.

#### 2.5.3 Post-intervention core data collection

Post-intervention data were collected after the intervention was introduced, which included CHV and private clinic HW mRDT interpretation results entered into the app, PCR results based on DBS collection, and interpretation results of mRDT images collected through the app, as determined by an independent, objective review panel (Panel Readers). Health workers also completed the same standard forms, including the MOH form and suspected malaria referral register form, as part of their normal workflow. Post-intervention data collection was set to reach completion after 2,080 mRDT interpretations were received from CHVs and 3,264 mRDT interpretations were received from private clinic HWs.

#### 2.5.4 Endline health worker survey

After post-intervention data collection, with a total app usage time of 13 weeks (June 7 to September 6, 2021), CHVs and private clinic HWs completed an endline survey, which included questions on their app experiences, malaria implementation knowledge, and visual acuity updates (S1 File). The survey was piloted for usability with a random sample of health workers before deployment. CHEWs also completed an endline questionnaire which collected their demographic information and experiences supervising CHVs. A copy of the baseline and endline data is provided as a S3 File.

### 2.6 *HealthPulse* app flow overview

To begin testing a new patient, health workers wrote the patient’s name and the current date on their MOH case reporting and DBS forms, then entered the same information into the app. The health worker input the patient’s demographic information and clinical symptoms, which included gender, weight/age category, body temperature (Celsius), and use of fever-reducing medication before the visit. Health workers were asked to check any symptoms the patient was experiencing from a provided list. The health worker then confirmed they were using an SD Bioline malaria Ag *P*.*f* mRDT, referenced as SD Bioline *P*.*f* throughout the text, or indicated if they would use a different brand for the test, such as AccessBio’s CareStart malaria *P*.*f*, which was another common mRDT used by health workers in Busia at the time. As different mRDTs could require different administration steps (for example, the CareStart test requires two diluent drops vs. four as indicated in the SD Bioline in-app instructions) [32], photos uploaded with an alternate mRDT were flagged during the data review process.

After prompting the health worker to check the mRDT’s expiration date, they were guided through digital use instructions (specific to the SD Bioline *P*.*f* procedures) to administer the test, including guidance to add four diluent drops to the mRDT to yield an accurate result. The health worker used the 15-minute in-app mRDT processing timer to ensure adherence to the minimum processing time. Data were also captured to determine if they read the result during the intended 15–30 minute window after the test began processing. After the test finished processing, the health worker took a photo of the mRDT using the app, which included an on-screen sample image with tips to help them take a good photo. The app processed the photo to determine whether it was of sufficient quality (e.g., checks to ensure the expected mRDT was present in the photo and that the mRDT was properly scaled within the photo frame) and asked the health worker to retake the photo if needed. Once the photo was accepted by the app, the health worker was guided through steps to interpret the mRDT. They were asked to identify whether the mRDT had only a control (C) line (indicating a negative result), had a test (*P*.*f*) line and a control (C) line (indicating a positive result), or whether the control (C) line was absent (indicating an invalid result).

The app then asked the health worker if they were dispensing antimalarials to the patient and if so, to state the doses dispensed of Artemether-lumefantrine (AL) (6, 12, 18 and 24 tablets at 20/120mg) based on the patient’s age/weight category. If the health worker’s dispensing practices contradicted the standard of care (such as dispensing an antimalarial for an individual with a negative mRDT), the health worker was asked to state a reason why. If the health worker dispensed another type of ACT, this was not documented through the app. Lastly, the worker was directed to prepare the DBS sample to be sent to a lab for additional testing, review the test results summary, and provide any additional comments on their testing experience. Refer to S1 Fig for sample app images.

Availability and supply of mRDTs and AL packets were documented through the app’s “supply bag” feature. Whenever health workers received supplies, they were required to manually update their supply levels through this feature. The number of mRDTs used and ACTs dispensed was automatically removed from the supply bag after each encounter with the app. If stock was lost during the study or stock was used without a complete encounter, the health worker was able to manually update this change.

The app had exactly the same functionality when working online (had an active internet connection) and offline (no active internet connection), meaning health workers were able to fully complete their test and treatment procedures and capture mRDT photos while offline. Data collected when the app was offline was automatically uploaded when the mobile device established an online connection without requiring any additional steps by the health worker. Only uploaded data were available for review and analysis by study stakeholders.

### 2.7 Study outcome measures and analysis

The primary objective of this study was to assess the feasibility of CHVs and private clinic HWs using *HealthPulse* in a low-resource setting. Feasibility was assessed through a range of routine and survey data, chosen to provide a breadth of metrics targeting the app’s usability and functionality from a user perspective. Routine *HealthPulse* process data included the quality of and access to an internet connection, time spent on instructional screens, result read window compliance (time between the start of the app’s mRDT processing timer and first photo upload, which measures whether mRDTs are interpreted in the appropriate time frame), encounter duration (time between starting a new encounter (starting from the home screen) and uploading a final, accepted photo). The quality of uploaded images was analyzed by Panel Readers, and counts were generated of the number of uninterpretable images, invalid cassettes, false negatives, and false positives. Instances of the same mRDT cassette photographed by a health worker were detected by identifying physical artifacts on the cassette, such as unique dents/scratches, and blood/diluent stains on the plastic cassette and/or diagnostic zone. Survey data included health worker perspectives on the app’s usability, malaria implementation knowledge. Personal phone ownership and use data were also captured to understand the compatibility of the app with different smartphone models and the familiarity of health workers with using other smartphone apps. Malaria implementation data was analyzed before and after the intervention was introduced. This included multiple choice questions on knowledge of the number of diluent drops required for proper mRDT function, minimum and maximum wait times for mRDT interpretation, the meaning of a visible *P*.*f* line only, as well as the interpretation of a standard set of sample mRDT images with positive, negative, and invalid results.

The secondary research objective was to analyze the acceptability of *HealthPulse* among health workers, investigating whether the app could be easily integrated into health workers’ standard workflow, and assessing if health workers felt that the app provided additional support for mRDT testing. Acceptability was analyzed by comparing responses between the baseline and endline health worker experience surveys, specifically questions on the app’s content, desired future features of the app, and health workers’ general impressions of participating in the study. User experience surveys used a mix of question types, including four-scale Likert, multiple choice, “select all that apply”, and open-ended responses. The level of respondent agreement to Likert statements was calculated by considering the proportion of “strongly agree” and “somewhat agree” responses among all responses (which also included “somewhat disagree” and “strongly disagree”). CHEW endline survey data were analyzed to understand supervisor perspectives of the app’s feasibility and acceptability among CHVs. Additional data on acceptability was analyzed through a follow-up qualitative study with results presented elsewhere.

Baseline visual acuity was assessed by training staff using the Jaeger Eye Test, a test of nearsightedness and reading ability which provided insight on the health workers’ ability to read the mRDT results [33]. During the endline survey, health workers were asked if they started wearing glasses or contacts during the study period.

All data collected on paper, such as baseline and endline surveys, were transcribed into Survey CTO and aggregated in Audere’s database along with process data collected through the app. For analysis, data were downloaded from Audere’s online database and analyzed using Microsoft Excel (Version 2201 (14931.20120)) and STATA 17 (StataCorp. 2021. Stata Statistical Software: Release 17. College Station, TX: StataCorp LLC). Duplicate (previously photographed) mRDTs were removed for calculations after the summary statistics on app usage were presented. Chi-squared tests (for categorical data) were run to determine statistical significance between baseline and endline results, with statistical significance set at p < 0.05 or at a 95% confidence interval (CI). Tables and figures were created to show comparisons between baseline and endline results and present trends in routine process data collected through the app.

### 2.8 Data monitoring, storage, and privacy

Data monitoring took place multiple times during the study. CHVs were first evaluated on how well they followed the study protocol through regular monitoring of a dashboard tracking app usage, mRDT data, and patient demographics. PS Kenya conducted weekly supervision checks and monthly review meetings with their field officers and research assistants, which allowed progress to be measured and monitored. PS Kenya, with support from Audere, performed data monitoring checks throughout the program to ensure accurate transcription and that app data was properly matched to MOH form data. Once all app data was collected, mRDT images were sent to the Panel Readers, an organization external to Audere which performed an independent, objective review and interpretation of mRDT images. These Panel Reader mRDT interpretations were compared to health workers interpretations entered into the app. All data collected through *HealthPulse* was stored securely using a HIPAA-compliant Amazon Web Services (AWS) cloud instance managed by Audere.

### 2.9 Supplies

In Kenya, roughly 60% of malaria commodities are procured by the Global Fund, while 40% are purchased directly by the government. Typically the MOH procures SD Bioline *P*.*f* mRDTs which are supplied to both the CHVs and private clinics. However, when supply challenges exist, the private clinics augment supply by purchasing mRDTs from commercial distributors. For this study, 90% of the mRDTs were purchased by Audere and PS Kenya to ensure access to mRDTs during the study, while the MOH provided the remaining 10% of tests. Other supplies came directly from the clinics’ normal supply chain, which included the AL delivered to patients and the case reporting forms. Prior to the study, stock shortages had led to mainly CareStart mRDTs being used in the clinics. Health workers began using the supplied SD Bioline *P*.*f* mRDTs again during the study and only used an alternate brand of mRDT when stockouts occurred.

### 2.10 Ethical considerations

Institutional review board (IRB) approval was received from AMREF Health Africa Kenya before the study began (Ref: AMREF-ESRC P897/2020). Prior to the baseline surveys, written informed consent was provided by all CHVs, private clinic HWs, and CHEWs. For patients, written informed consent was obtained for those 18 years or above. Verbal assent plus written parental informed consent was obtained for those ages 10 to less than 18 years. Consent and assent of patients enabled the study team to collect personally identifiable information used to match data between sources (e.g. MOH forms, DBS cards, and app collected data) and to collect a DBS sample used for study procedures.

All health workers received fair compensation during their participation in the study. CHVs were provided with 2000 Kenyan shillings (KSH) per month to compensate for necessary training travel and meals, CHEW/supervisor visits, and related expenses during the study. Both CHVs and private clinic HWs were compensated with data/airtime bundles and personal protective equipment (PPE) to perform testing. Patients did not receive compensation for their participation in the study. Additionally, all patients were guaranteed equal care regardless of their decision to participate. Information regarding the ethical, cultural, and scientific considerations specific to inclusivity in global research is included as a S2 File.

## 3. Results

### 3.1 Health worker demographics

A total of 223 health workers participated for the full study period, which included 200 CHVs and 23 private clinic HWs (nurses, clinical officers, and lab technicians). The highest number of CHVs (39.0%) were recruited from the Teso South sub-county while the largest percentage of private clinic HWs (30.4%) were recruited from the Teso North sub-county. Most CHVs (67.0%) volunteered at a dispensary, and 96.0% of the facilities CHVs volunteered at were government-owned. Most private clinic HWs worked at clinic-level facilities (69.6%) and locations under for-profit private ownership (73.9%). At enrollment, CHVs had a mean of 12.3 years (SD = 5.7) of experience as a health volunteer and a mean of 4.4 years (SD = 3.19) working in malaria. In comparison, the private clinic HW cohort had a higher mean years of health worker experience (M = 13.9, SD = 15.1) and a higher mean years working in malaria (M = 9.0, SD = 11.9), with a wider range for both categories.

The average age of CHVs was 46.8 years (SD = 9.6) while the average age of private clinic HWs was 39.7 years (SD = 16.1). Private clinic HWs completed more years of education at the time of enrollment compared to CHVs, with 100% of private clinic HWs having completed a college education or above compared to only 6.0% of CHVs. Approximately two-thirds of CHVs were female (68.5%) compared to 52.2% of private clinic HWs. The preferred reading language for most health workers was English (78.0%) while the preferred speaking language was Kiswahili (82.5%). Most health workers (67.7%) had average or better near vision at the start of the study. Full CHV and private clinic health worker demographics are presented in S2 Table.

Overall, 45 CHEWs were sampled throughout the five sub-counties. CHEWs were most commonly from Teso South (37.8%), female (66.7%), between 30–39 years of age (48.9%), and educated at a college level or higher (97.8%). While the years of experience working as a CHEW ranged from one year to more than six years, over half (53.3%) had between one to two years of experience working in malaria specifically. Full details of CHEW demographics are outlined in S3 Table.

### 3.2 Baseline health worker survey

#### 3.2.1 Phone use

Health workers used a variety of personal phone models and brands in the study. Overall, 86 different Android phone models were used across 18 brands, and most encounters (49.5%) were uploaded using phones that had a five megapixel camera. The top four phone brands and models used by health workers in the study were Safaricom Neon Ray (Neon Ray Pro and Neon Ray models) and Tecno (Tecno S6S and Tecno F1 models). Most encounters were uploaded using phones that were only a few years old, with 51.3% of encounters using phones released between 2018–2020 (the three years preceding the 2021 study). At baseline, 70.3% of health workers felt very comfortable using a smartphone, 28.3% felt somewhat comfortable, and 1.4% did not feel very comfortable. The most common apps used by health workers at the time of the study were SMS text messages (83.1%), WhatsApp (75.3%) and Facebook (47.0%). Less than 10% of health workers used other social media apps including Twitter, Instagram, or TikTok.

Results on app use and internet connectivity varied among health workers. While 30.6% (1,702/5,572) of photos (including photo retakes) were uploaded within an hour, it was common for health workers to lack consistent internet connectivity, with the remaining test data uploading once a connection was available. Offline use resulted in a slight delay of data availability to the study team, but not data loss. Even when the health worker was offline, the app was fully functional, confirming photo quality and capturing app usage details.

#### 3.2.2 Baseline attitudes to an mRDT support app

Most CHVs and private clinic HWs responded positively toward the idea of using an mRDT reader app. There was at least 94% agreement with eight out of twelve supportive statements related to app design and use, including the utility of digital instructions and an mRDT timer; the ability to share data with supervisors electronically; time saved from not having to use paper routine monitoring forms; and the ability to capture treatment information.

Fewer than 80% of health workers agreed on four survey topics, with differences in CHV and private clinic HW responses. For example, while 79.0% of CHVs agreed that it would be helpful for their supervisor to see their work more quickly, only 60.9% of private clinic HWs responded similarly. Additionally, while 73.9% of private clinic HWs agreed that an mRDT image captured through the app would prove that they completed the test, only 68.5% of CHVs agreed. Fewer than 80% of health workers agreed that they would be confident in their mRDT results being properly saved by an app. Details regarding health worker attitudes toward the app are presented in Table 1.

**Table 1:** Baseline health worker attitudes towards an mRDT reader app.

Baseline health worker attitudes toward an mRDT reader app			
Survey Questions	Total (N = 223)	CHVs (N = 200)	Private clinic HWs (N = 23)
	Frequency Agree* (%)	Frequency Agree (%)	Frequency Agree (%)
I would find it useful to have an app with digital instructions to show me how to do an mRDT.	219 (98.2)	196 (98.0)	23 (100.0)
I would prefer using an app to enter data versus paper instructions and paper tracking sheets.	218 (97.8)	195 (97.5)	23 (100.0)
I would find it helpful to have an app tracking when an mRDT is ready to be read.	219 (98.2)	197 (98.5)	22 (95.7)
I believe using an app vs. using paper instructions and paper tracking sheets could save me time when working with community members.	210 (94.2)	189 (94.5)	21 (91.3)
I would find it helpful if an app captured treatment information (such as the medications I give to community members).	219 (98.2)	197 (98.5)	22 (95.7)
I would find it useful if my supervisor were able to access my mRDT results and malaria treatment data on a regular basis via an app, versus waiting for me to send in my paper form.	218 (97.8)	196 (98.0)	22 (95.7)
If an app provided the mRDT interpretation directly (instead of me needing to indicate whether it was positive or negative), I would likely trust that result.	214 (96.0)	193 (96.5)	21 (91.3)
It would be useful for an app to provide more information about malaria testing happening in my area and surrounding areas.	215 (96.4)	194 (97.0)	21 (91.3)
The image proves I did an mRDT test for the community member.	154 (69.1)	137 (68.5)	17 (73.9)
The picture can sometimes help me see if the test result was positive or negative.	170 (76.2)	155 (77.5)	15 (65.2)
I am confident that my mRDT results are being properly saved.	167 (74.9)	149 (74.5)	18 (78.3)
I like that my supervisor can more quickly see the efforts of my work.	172 (77.1)	158 (79.0)	14 (60.9)

* “Frequency agree” is the number (%) of respondents who “strongly agreed” or “somewhat agreed” to each statement. Other response options were “somewhat disagree” and “strongly disagree.”

#### 3.2.3 Baseline mRDT implementation knowledge

Health worker mRDT implementation knowledge at baseline had gaps. Only 8.1% of health workers correctly specified that four diluent drops were necessary for the SD Bioline *P*.*f* mRDT to function properly. Additionally, only 30.0% of CHVs and 39.1% of private clinic HWs correctly marked the minimum wait time before reading the mRDT result as 15 minutes. There was also variation in health worker responses for the maximum wait time. While 10.3% of health workers accurately marked 30 minutes, most health workers (60.5%) selected 10 minutes for their response.

When asked to interpret sample mRDT images (S2 Fig), more than 95% of health workers correctly interpreted the strong positive, medium positive, and invalid mRDTs while 85.2% of health workers correctly interpreted the negative mRDT image. However, only half of health workers (50.2%) accurately identified a light *P*.*f*. line on the sample mRDT image as a positive, and correct interpretation of a very faint *P*.*f*. line was even lower (12.6%). Full baseline mRDT implementation knowledge results are shared in Table 5.

### 3.3 Health worker app use

Health workers administered 5,278 mRDTs using the guidance provided by *HealthPulse* during the post-intervention phase (Table 2). Each CHV administered a mean of 11.3 mRDTs (SD = 4.5) during the study, while each private clinic HWs administered a mean of 130.8 mRDTs (SD = 107.0). During analysis, 879 mRDTs were flagged as duplicates (the same mRDT cassette appearing in photos for separate patient encounters and uploaded by the same health worker), which comprised 16.7% of all mRDTs administered in the study. After removing these duplicates, each CHV administered a mean of 10.2 mRDTs (SD = 4.4) during the study, while each private clinic HWs administered a mean of 102.3 mRDTs (SD = 101.9).

**Table 2:** Summary of health worker app usage.

Health Worker Group	Number of mRDTs administered using *HealthPulse*	Mean	Standard Deviation (SD)	Median	Range (Lower- Upper)
*With Duplicate mRDTs*					
CHV	2,269	11.3	4.5	11	2–28
Private clinic HWs	3,009	130.8	107.0	87	23–377
All Health Workers	5,278	23.7	49.8	12	2–377
*Without Duplicates mRDTs*					
CHV	2,047	10.2	4.4	9	2–28
Private clinic HWs	2,352	102.3	101.9	58	17–358
All Health Workers	4,399	19.7	42.8	10	2–358

### 3.4 Process controls

**Fig 4:**
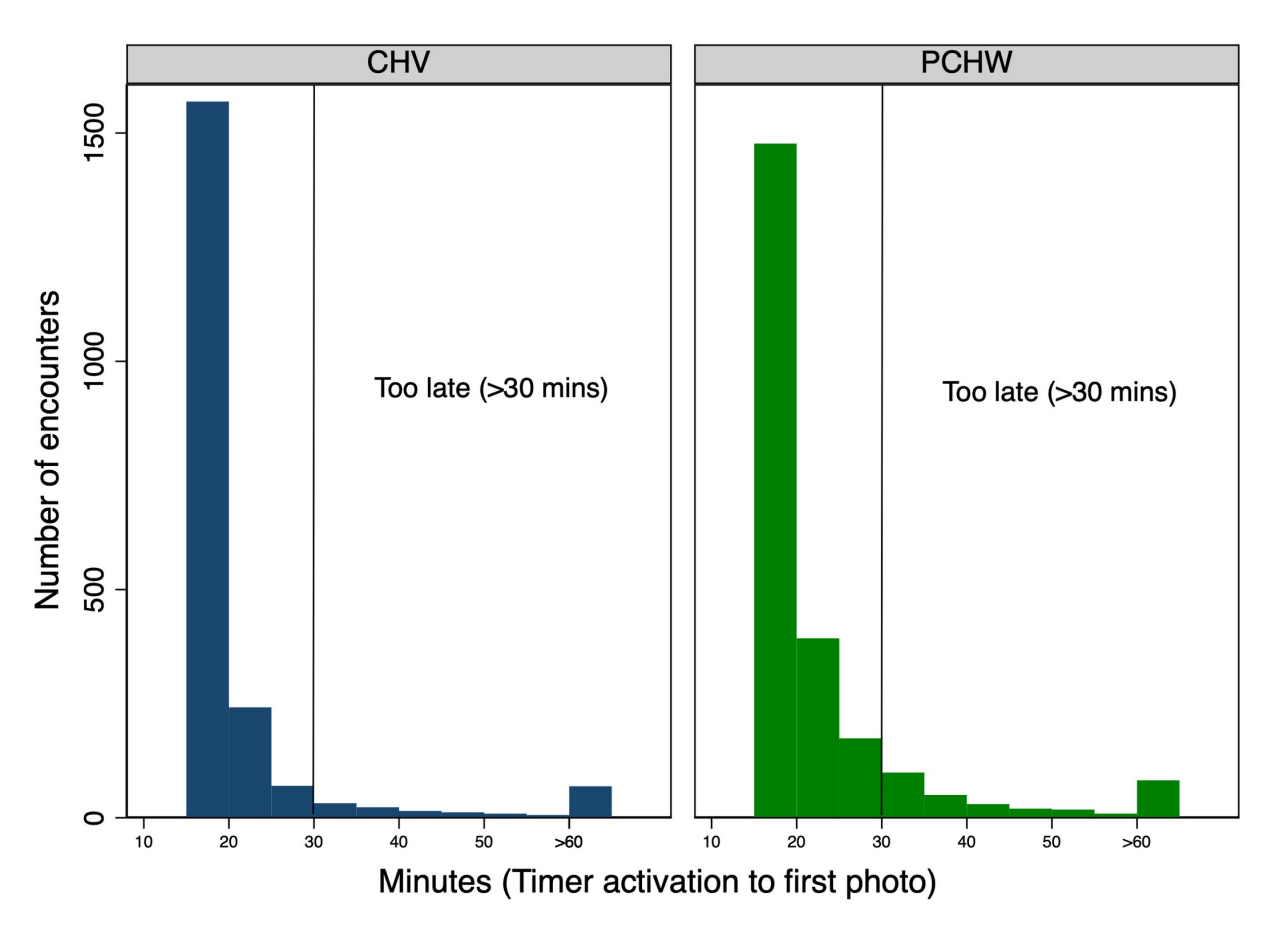
Read window compliance: Time from the activation of the HealthPulse timer to the mRDT being photographed, by type of health worker.

**Fig 5:**
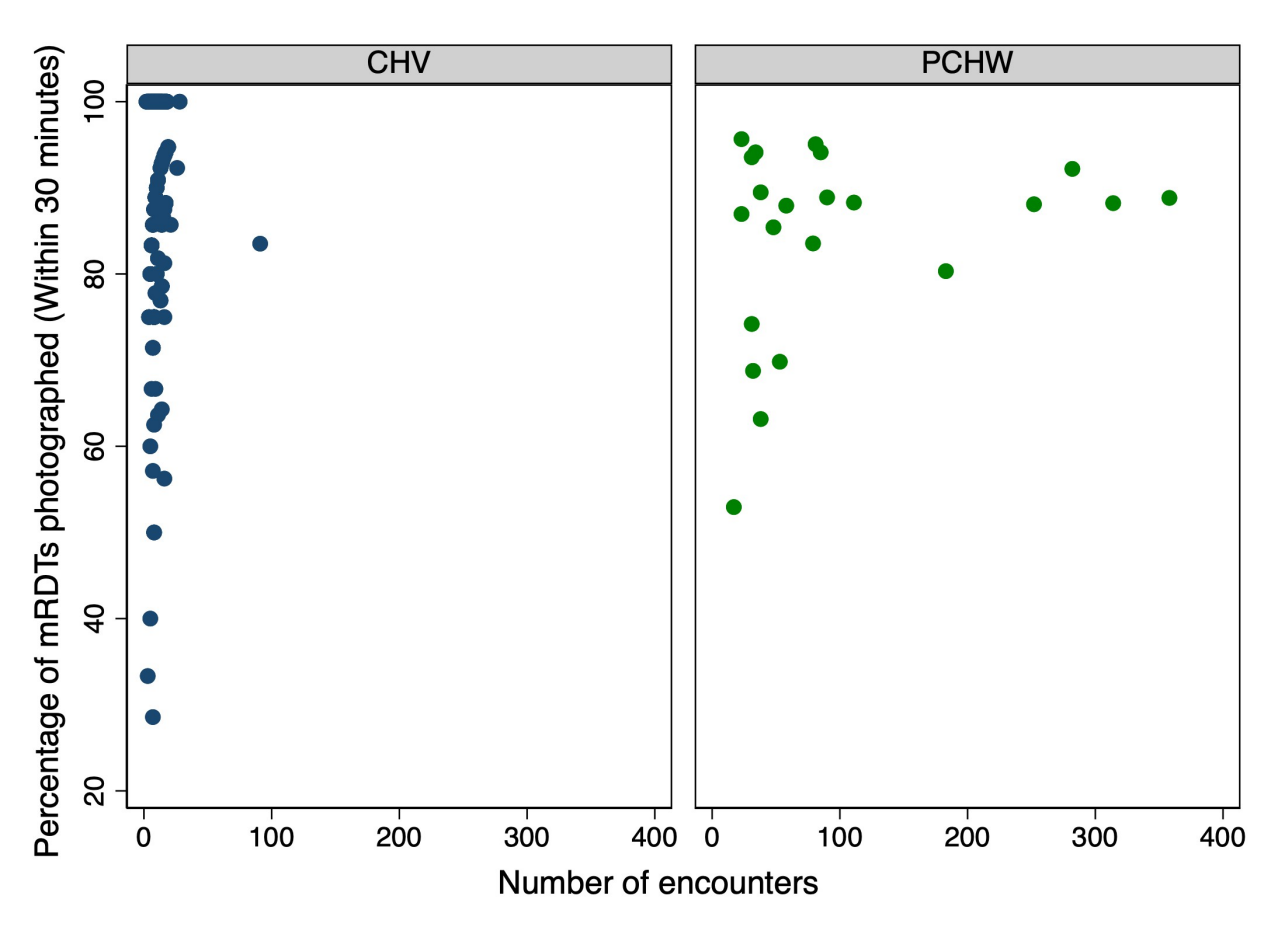
Read window compliance: Percentage of mRDTs photographed within 30 minute interpretation window, by type of health worker.

Among the final mRDT photos submitted through the app, 91.4% (4,021/4,399) were accepted during the first submission, meaning no photo retake was requested. mRDT read window compliance showed that 89.2% of health workers’ initial mRDT encounters (3,925/4,399) were photographed within the 30-minute recommended timeframe, with 7.3% of encounters (323/4,399) photographed 30–60 minutes after the mRDT was activated and 3.4% (151/43,99) photographed after one hour (Fig 4). As the app did not allow health workers to take a photo of the mRDT before the manufacturer’s recommended 15-minute waiting time, there were no images captured early. It was found that health workers did not consistently time photographing the mRDT across all of their own patient interactions. Some health workers waited longer to photograph the mRDT, with only 53.5% of CHVs (107/200) and 0.0% of private clinic HW (0/23) photographing *all* their mRDTs within the manufacturer’s recommended 30-minute interpretation window from activation (Fig 5). Separately, there were seven encounters (0.2% of all encounters) among six health workers (two private clinic HW and four CHVs) where the mRDT photo showed an invalid cassette (no control line).

### 3.5 mRDT administration and interpretation

The quality of health worker mRDT interpretation varied among health workers when compared to Panel Reader interpretation. For example, private clinic HWs had more false negatives (15/2,352) while CHVs had more false positives (83/2,047). The Panel Reader interpretation also revealed that while three health workers contributed to 12.6% (18/143) of the incorrect interpretations, the remaining interpretation errors were spread evenly among workers. Overall, 7.6% of all uploaded mRDT photos were uninterpretable by the Panel Reader mRDT interpreters. The most common issues that prevented interpretation included having excessive blood/diluent stains in the diagnostic area (50.3%), a shadow (44.2%), or a skewed image (41.5%).

While 89.6% of all encounters used the SD Bioline *P*.*f*. mRDT, there were additional brands of mRDTs flagged in photos during the review. For example, 2.8% of encounters had a CareStart mRDT in the final, uploaded image. During the study, CHVs uploaded more encounters with a CareStart mRDT (5.7%, n = 116/2,047) than private clinic HWs (0.3%, n = 6/2,352).

### 3.6 ACTs dispensed

**Fig 6:**
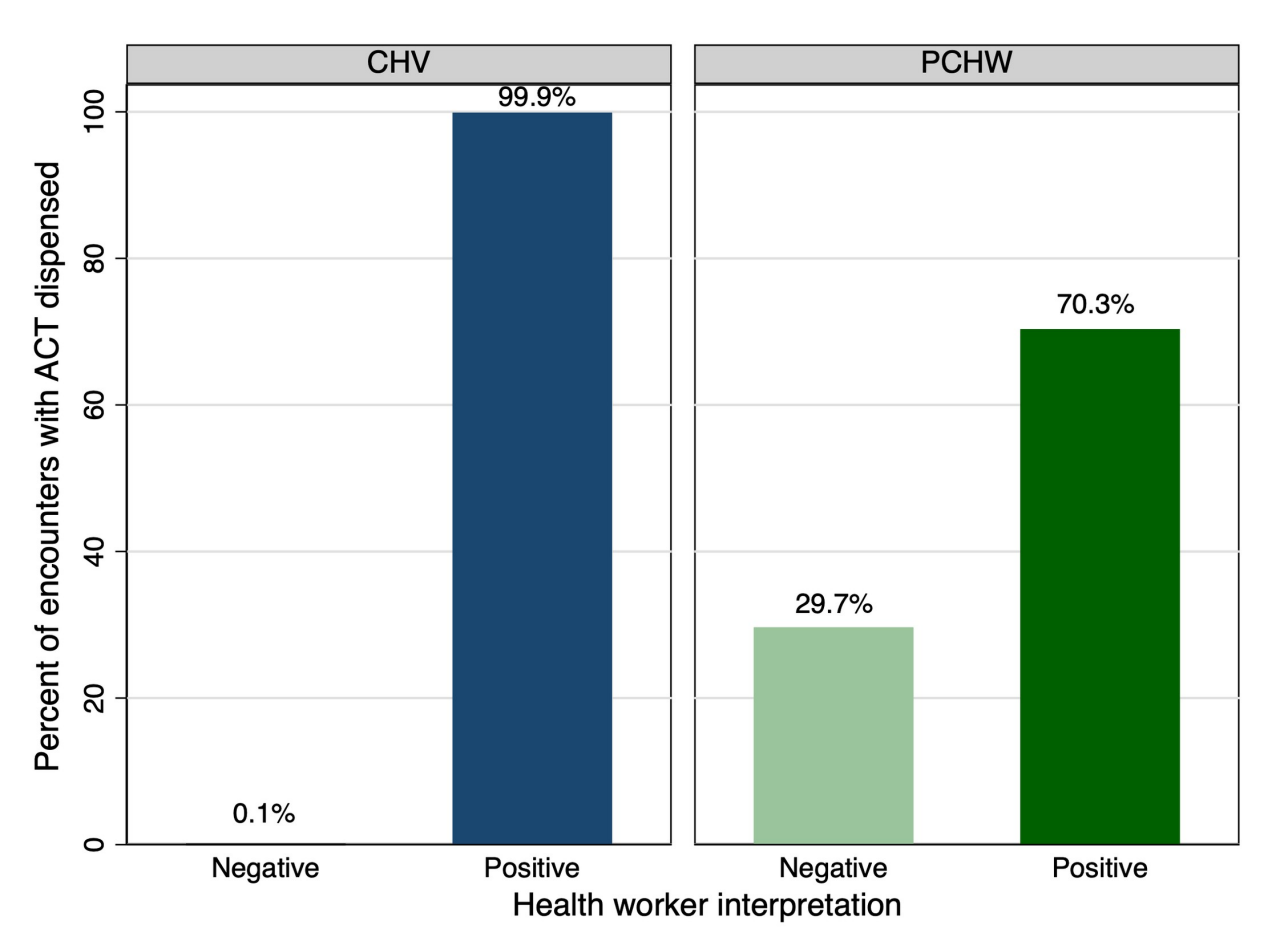
Percentage of encounters with ACTs dispensed, by type of health worker and by mRDT result.

For 154 encounters, a health worker provided medication to their patient even though the health worker interpreted the mRDT as negative (3.5% of encounters) (Fig 6). These encounters were distributed among one CHV (responsible for one of these instances) and five private clinic HWs. One private clinic HW was responsible for 68.2% of these encounters (105/154). When providing reasons for why medication was prescribed for a negative test result, this private clinic HW shared that their microscopy results were positive, although it is unclear if microscopy results were actually available to this health worker at the time of this response. Additional reasons for dispensing ACTs for negative mRDT results were that the patient presented with what the health worker deemed to be clear malaria symptoms (such as fever), the patient requested or demanded medication, or the health worker felt obligated to provide the patient with the medication. Instances where an mRDT was positive, but no medication was prescribed were due to the medication not being available at the time of the patient encounter. A copy of the app data used for the results in sections 3.3 and 3.4 is provided as a S4 File.

### 3.7 Endline health worker survey

#### 3.7.1 Endline *HealthPulse* experience

In the endline health worker survey, 99.6% of health workers reported the app to be useful and 90.1% reported that the app was easy to use. Both CHVs and private clinic HWs felt highly confident in reading the mRDT results using the app and 96.4% of health workers felt that the app provided clear and helpful instructions. Most CHVs (93.0%) felt that the app would reduce the time required for data reporting and 91.5% preferred submitting data via the app to paper reporting, while 91.3% of private clinic HWs felt similarly on both topics. Health workers handled their mobile device well; with only a few health workers sharing challenges of keeping the phone clean and charged and finding a suitable surface for taking photos. Most health workers agreed that the app’s instructional materials were helpful, including the timer (98.7%), the example images for interpreting the mRDT (97.3%), and the ability to track treatment provided to patients in the app (97.8%). Health workers’ perceptions of the in-app supply bag feature were also measured in the endline survey, in which 99.1% of health workers agreed that the supply bag feature was easy to use.

While health workers valued the app’s features, some shared difficulties with capturing photos of the mRDTs. Most health workers (71.7%) indicated on the endline survey they had difficulties getting a good photo at some point in the study, although roughly one quarter of health workers (27.8%) only had difficulties the first few times they took the photo. During the endline survey, 32.3% (72/223) of health workers recalled being prompted to retake a photo during one or more of their encounters. However, not all health workers followed the in-app retake guidance every time. Among those prompted by the app to retake a photo, health workers indicated they opted to use their current image for reasons such as feeling that their first photo was good enough (61.1%), feeling that retaking the photo didn’t matter (12.5%), having no time for a retake (5.6%), or having an unsuccessful photo even after the retake (33.3%). When taking photos, just under half of the health workers captured them inside using sunlight from a window (46.2%) while others used overhead lights or a lamp (24.7%) or captured photos outside (28.7%).

Health workers also provided strong support for future additions to the app. For example, 100.0% of private clinic HWs and 98.5% of CHVs agreed that it would be useful for their supervisor to have access to their app results. Also, 91.9% of health workers felt that it would be useful if the app provided an interpretation of the mRDT result. Lastly, 92.8% of health workers agreed that it would be useful for the app’s results to inform a malaria incidence dashboard. Full results of the endline survey are presented in Table 3.

**Table 3:** *HealthPulse* app experience among CHVs and private clinic HWs.

*HealthPulse App User Experience of CHVs and Private clinic HWs*			
Survey Question	Total (N = 223)	CHVs (N = 200)	Private clinic HWs (N = 23)
	N (%)	N (%)	N (%)
***Project Impressions***			
Overall impression that app is useful [Likert]	222 (99.6)	199 (99.5)	23 (100.0)
Easy to use the app [Likert]	201 (90.1)	180 (90.0)	21 (91.3)
Information in app was trustworthy [Likert]	217 (97.3)	194 (97.0)	23 (100.0)
Instructions were clear and helpful [Likert]	215 (96.4)	192 (96.0)	23 (100.0)
App would save time compared to paper when working with community members [Likert]	207 (92.8)	186 (93.0)	21 (91.3)
Prefer app to paper instructions/tracking [Likert]	204 (91.5)	183 (91.5)	21 (91.3)
App improved skills of mRDT administration/interpretation [Likert]	212 (95.1)	191 (95.5)	21 (91.3)
Felt confident in reading mRDT results [Likert]	219 (98.2)	196 (98.0)	23 (100.0)
***App Usability***			
Supply bag was easy to use [Likert]	221 (99.1)	198 (99.0)	23 (100.0)
Able to see app content easily [Likert]	185 (83.0)	162 (81.0)	23 (100.0)
No challenges with using a mobile device	142 (62.0)	129 (64.5)	13 (56.5)
Challenge: Hard to keep phone clean [Select all that apply]	26 (11.7)	23 (11.5)	3 (13.0)
Challenge: Hard to keep phone charged [Select all that apply]	42 (18.8)	39 (19.5)	3 (13.0)
Challenge: Hard to handle both the phone and test supplies [Select all that apply]	37 (16.6)	32 (16.0)	5 (21.7)
Challenge: Hard to find a flat surface to put phone on [Select all that apply]	20 (9.0)	18 (9.0)	2 (8.7)
***mRDT Usage***			
Health worker did not recall using a non-study mRDT brand during the study	134 (60.1)	115 (57.5)	19 (82.6)
Reason health worker used a non-study mRDT: SD Bioline *P*.*f* was not available	29 (13.0)	29 (14.5)	0 (0.0)
Reason health worker used a non-study mRDT: Other mRDTs were in stock	28 (12.6)	24 (12.0)	4 (17.4)
Reason health worker used a non-study mRDT: other reason (e.g. by mistake)	4 (1.8)	4 (2.0)	0 (0.0)
***App Content***			
Instructional pictures were useful [Likert]	206 (92.4)	187 (93.5)	19 (82.6)
Instructional text was useful [Likert]	206 (92.4)	186 (93.0)	20 (87.0)
Timer helped to know when mRDT was ready [Likert]	220 (98.7)	197 (98.5)	23 (100.0)
Example mRDT images were useful when interpreting result [Likert]	217 (97.3)	195 (97.5)	22 (95.7)
Capture of treatment information was helpful [Likert]	218 (97.8)	195 (97.5)	23 (100.0)
Utility of saved mRDT image: Proves test for the community member [Select all that apply]	158 (70.9)	138 (69.0)	20 (87.0)
Utility of saved mRDT image: Image can help determine interpretation [Select all that apply]	196 (87.9)	175 (87.5)	21 (91.3)
Utility of saved mRDT image: Provides confidence that results are being saved [Select all that apply]	176 (78.9)	154 (77.0)	22 (95.7)
Utility of saved mRDT image: Supervisor can more quickly see work efforts [Select all that apply]	159 (71.3)	143 (71.5)	16 (69.6)
***Photos***			
Getting a good photo was difficult at one or more times during the study [Likert]	160 (71.7)	144 (72.0)	16 (69.6)
Health worker only had difficulties during the first few times they took the photo [Likert]	62 (27.8)	53 (26.5)	9 (39.1)
Health worker recalled skipping one or more photo retake requests from the app	72 (32.3)	65 (32.5)	7 (30.4)
**Reason retake request was skipped: Did not have time [Select all that apply]	4 (5.6)	4 (6.2)	0 (0.0)
**Reason retake request was skipped: Feeling the retake photo did not matter [Select all that apply]	9 (12.5)	7 (10.8)	2 (28.6)
**Reason retake request was skipped: Tried retaking but photo did not look better [Select all that apply]	24 (33.3)	22 (33.8)	2 (28.6)
**Reason retake request was skipped: Felt the photo was already good enough [Select all that apply]	44 (61.1)	40 (61.5)	4 (57.1)
Typically took photos inside using sunlight from window [Multiple choice]	103 (46.2)	90 (45.0)	13 (56.5)
Typical took photos inside using overhead lights or lamp [Multiple choice]	55 (24.7)	47 (23.5)	8 (34.8)
Typically took photos outside using sunlight [Multiple choice]	64 (28.7)	62 (31.0)	2 (8.7)
Typically used other lighting not listed [Multiple choice]	1 (0.04)	1 (0.50)	0 (0.0)
***Future Features***			
Supervisor access to results would be useful [Likert]	220 (98.7)	197 (98.5)	23 (100.0)
App-provided interpretation would be useful [Likert]	205 (91.9)	186 (93.0)	19 (82.6)
A local malaria incidence dashboard would be useful [Likert]	207 (92.8)	185 (92.5)	22 (95.7)
***Visual Acuity***			
Started wearing contacts/glasses during study [Multiple choice]	16 (7.2)	14 (7.0)	2 (8.7)

*Likert questions are presenting the % of respondents that selected one of two positive responses

**Calculated among health workers who recalled being requested to retake a photo in the app (n = 72)

#### 3.7.2 Endline mRDT implementation knowledge

The endline survey demonstrated shifts in the health workers’ mRDT implementation knowledge. Health workers improved their knowledge of the required number of diluent drops, as 56.1% of health workers correctly recalled four drops after the intervention compared to 8.1% before the intervention (p = 0.002). However, responses clearly differed between CHVs and private clinic HWs, as 73.9% of private clinic HWs provided the correct answer after the intervention compared to 54.0% of CHVs. Knowledge of the minimum wait time before reading the mRDT also improved, with 68.6% of health workers choosing the correct answer of 15 minutes after the intervention, compared to only 30.9% of health workers before the intervention (p = 0.063). A slight improvement was made on the knowledge of the maximum wait time, with 15.2% of health workers correctly identifying 30 minutes after the intervention compared to 10.3% of health workers at baseline (p<0.001), although correct responses still remained low.

While reviewing sample mRDT images, CHVs and private clinic HWs made slight improvements from the baseline survey when correctly identifying strong positives and negatives, although health workers performed worse compared to baseline when identifying medium, light, and very faint *P*.*f*. lines as true positives. For example, while only 2.7% of health workers correctly identified sample mRDT images with a very faint line as positive after the intervention, 12.6% of health workers correctly identified these mRDT images as positive before the intervention took place (p = 0.305). The percentage of health workers who accurately identified an invalid mRDT and a strong positive mRDT from the sample images remained high after using the app. Full endline malaria implementation knowledge results are presented in Table 4.

**Table 4:** Malaria implementation knowledge among CHVs and private clinic HWs (baseline and endline comparison).

mRDT Implementation Knowledge									
Survey Questions	Total (N = 223)			CHVs (N = 200)			Private clinic HWs (N = 23)		
	Baseline (%)	Endline (%)	p-value	Baseline (%)	Endline (%)	p-value	Baseline (%)	Endline (%)	p-value
**SD Bioline *P*.*f* Diluent Drops** *									
4 drops (correct)	18 (8.1)	125 (56.1)	p = 0.002	15 (7.5)	108 (54.0)	p = 0.002	3 (13.0)	17 (73.9)	p = 0.426
Less than 4 drops	199 (89.2)	98 (43.9)		180 (90.0)	92 (46.0)		19 (82.6)	6 (26.1)	
More than 4 drops	6 (2.7)	0 (0.0)		5 (2.5)	0 (0.0)		1 (4.3)	0 (0.0)	
**Minimum Wait Time Before Reading mRDT** *									
15 min (correct)	69 (30.9)	153 (68.6)	p = 0.063	60 (30.0)	133 (66.5)	p = 0.178	9 (39.1)	20 (87.0)	p = 0.344
Less than 15 minutes	13 (5.8)	3 (1.3)		9 (4.5)	2 (1.0)		4 (17.4)	1 (4.3)	
More than 15 minutes	141 (63.2)	67 (30.0)		131 (65.5)	65 (32.5)		10 (43.5)	2 (8.7)	
**Maximum Wait Time for Reading mRDT** *									
30 min (correct)	23 (10.3)	34 (15.2)	p<0.001	20 (10.0)	29 (14.5)	p<0.001	3 (13.0)	5 (21.7)	p = 0.775
10 min	135 (60.5)	148 (66.4)		127 (63.5)	141 (70.5)		8 (34.8)	7 (30.4)	
20 min	36 (16.1)	20 (9.0)		29 (14.5)	18 (9.0)		7 (30.4)	2 (8.7)	
40 min	6 (2.7)	8 (3.6)		5 (2.5)	7 (3.5)		1 (4.3)	1 (4.3)	
50 min	19 (8.5)	13 (5.8)		18 (9.0)	5 (2.5)		1 (4.3)	8 (34.8)	
Don’t know	4 (1.8)	0 (0.0)		1 (0.5)	0 (0.0)		3 (13.0)	0 (0.0)	
**Meaning of P.f Line Only** *									
Invalid (correct)	140 (62.8)	116 (52.0)	p<0.001	121 (60.5)	98 (49.0)	p<0.001	19 (82.6)	18 (78.3)	p = 0.050
Negative	74 (33.2)	90 (40.4)		70 (35.0)	87 (43.5)		4 (17.4)	3 (13.0)	
Positive	7 (3.1)	17 (7.6)		7 (3.5)	15 (7.5)		0 (0.0)	2 (8.7)	
Don’t know	2 (0.9)	0 (0.0)		2 (1.0)	0 (0.0)		0 (0.0)	0 (0.0)	
**Interpretation of Results for Sample Images** **									
Saw a positive for a strong positive	216 (96.9)	220 (98.7)	p = 0.099	194 (97.0)	197 (98.5)	p = 0.094	22 (95.7)	23 (100.0)	NA
Saw a positive for a medium positive	212 (95.1)	194 (87.0)	p = 0.062	192 (96.0)	173 (86.5)	p = 0.111	20 (80.7)	21 (91.3)	p = 0.104
Saw a positive for a light positive	112 (50.2)	65 (29.1)	p = 0.190	105 (52.5)	54 (27.0)	p = 0.028	7 (30.4)	11 (47.8)	p = 0.303
Saw a positive for a very faint positive	28 (12.6)	6 (2.7)	p = 0.305	26 (13.0)	4 (2.0)	p = 0.109	2 (8.7)	2 (8.7)	p = 0.776
Saw a negative for a negative	190 (85.2)	205 (91.9)	p = 0.108	171 (85.5)	183 (91.5)	p = 0.069	19 (82.6)	22 (95.7)	p = 0.896
Saw an invalid for an invalid	213 (95.5)	211 (94.6)	p = 0.711	192 (96.0)	188 (94.0)	p = 0.704	21 (91.3)	23 (100.0)	NA

*Question format: Multiple choice

**Question format: Fill in the blank (positive, negative, or invalid) by matching sample mRDT images

### 3.8 CHEW experience

CHEWs also responded positively in their app surveys. Overall, 100.0% of CHEWs felt that the app was useful for CHVs, though only 60.0% felt that it was easy for CHVs to use. Like health workers, CHEWs felt strongly that the timer was an important feature, with 97.8% of CHEWs in agreement. Additionally, 97.8% of CHEWs felt that the app’s instructions were helpful and that the app improved CHVs’ administration skills. Additional details on the CHEW’s experiences are outlined in Table 5. Like CHVs and private clinic HWs, most CHEWs (95.6%) agreed that it would be useful for the app to provide an interpretation of the test to the health worker.

**Table 5:** *HealthPulse* app experience among CHEWs.

*HealthPulse* App Experience Survey for CHEWs (Endline) (N = 45)		
Survey Question*	Response	Frequency (%)
Overall impression that app is useful	Useful or very useful	45 (100.0)
Easy for CHVs to use the app	Easy or very easy	27 (60.0)
Information in app was trustworthy	Strongly or somewhat agree	45 (100.0)
Instructions were helpful	Strongly or somewhat agree	44 (97.8)
App improved skills of mRDT administration	Strongly or somewhat agree	44 (97.8)
App improved skills of mRDT interpretation	Strongly or somewhat agree	42 (93.3)
Prefer app over paper for progress tracking	Strongly or somewhat agree	44 (97.8)
Prefer app to paper for stock tracking	Strongly or somewhat agree	44 (97.8)
Timer helped to know when mRDT was ready	Strongly or somewhat agree	44 (97.8)
Feel good about the photo	Strongly or somewhat agree	45 (100.0)
Would like app to provide interpretation	Strongly or somewhat agree	43 (95.6)

*Question format: 4-point Likert

## 4. Discussion

mRDTs are a valuable resource for malaria case management, although misadministration and misinterpretation can reduce the effectiveness of the tool. To explore how a digital health solution may support health workers in correctly performing and interpreting these tests in low-resource settings, the team assessed the feasibility and acceptability of implementing *HealthPulse* among a cohort of CHVs and private clinic HWs in Busia County, Kenya. Through baseline and endline surveys, as well as process control data collected through the app, this study documented the *HealthPulse* user experience, measured changes to malaria implementation knowledge, and identified trends in app usage. Results showed that while the *HealthPulse* app shows potential as a supportive tool, adding and/ or updating features may further enhance health worker app use.

### 4.1 Feasibility

#### 4.1.1 Process measures

*HealthPulse* was compatible across a wide range of phone brands and models, highlighting its potential for use among different settings. However, the study noted some technical and user challenges during app use, such as smartphone ownership, which was a cause of health worker attrition during the study. Although global access to smartphone ownership is projected to rise both in Kenya [28,29] and globally [34], supplementation of smartphones may be necessary. Since this study involved health workers using personal mobile devices, their data/airtime costs for app use were covered. However compensating usage for a specific application is difficult when the device is being used for multiple purposes. Future app versions could consider zero rating the app through mobile carriers or setting up a reverse billing arrangement whereby app usage is billed back to the program through the app developer, rather than using health worker data bundles.

While the results of some photos (30.5%) were uploaded within an hour, most were not automatically uploaded until the phone was reconnected to the internet. Data upload delays may lead to barriers when the program use case depends on timely availability of data. However, compared to current paper procedures, especially for the CHVs whose data is typically only shared monthly, the app provided a more reliable, timely, and consistent data pathway. The app’s ability to collect data while offline was particularly important to support data collection in rural communities.

While process data showed that health workers did not spend a long time reading the instructional screen describing how to correctly administer an mRDT, most health workers agreed on the endline survey that the instructions were clear and helpful (96.4%), that the instructional pictures were useful (92.4%), and that the instructional text was useful (92.4%). Follow-up studies may explore whether health workers were relying on the instructional images, using them as a high level guide and skimming over the written instructions, or if they felt that they already had sufficient confidence in correctly administering their mRDT.

#### 4.1.2 Photo quality and content

Most photos uploaded through *HealthPulse* were of high quality and accepted on their first submission. While most health workers (71.7%) shared they had difficulties getting a good photo at some point during the study, 27.8% shared they only had difficulties during the first few times they took the photo.

Ensuring quality photo capture is necessary to harness the power of processing mRDT images using artificial intelligence (AI). A current version of the HealthPulse app includes additional image quality assessment features, including checks for lighting and blur, which were not assessed in this study. Additional photo capture guidance and best practices can also be provided in the app and during health worker app training.

#### 4.1.3 Read window compliance

The app did not enable health workers to submit a photo of the mRDT before the 15-minute processing timer was complete, with the aim of preventing mRDTs from being interpreted too early. While the majority of encounters were photographed quickly, 10.8% of encounters were not photographed within the test’s 30 thirty-minute read window, which may indicate that health workers also did not interpret the test properly within this recommended time frame. While the app did not enforce that the mRDT result be digitized within the read window (to enable data collection on this topic), current versions of *HealthPulse* offer an optional setting to require digitization of the result within the manufacturer’s declared read window.

Survey results showed that while most health workers (68.6%) selected the correct minimum wait time for reading an mRDT on the endline survey, only 15.2% selected the correct response for the maximum wait time. These mRDT implementation knowledge results highlight the need for additional training and education for health workers, as these knowledge gaps are among the many factors affecting mRDT performance in diagnosing malaria [35,36]. The *HealthPulse* team can consider ways to help health workers improve malaria diagnosis and best practices through future app enhancements to more deeply embed key administration and interpretation procedures and guidelines into the app workflow. In-country routine data reporting procedures could be streamlined by integrating the app into the health workers’ workflow, which can reduce the reliance on paper reporting and paper mRDT administration and interpretation materials.

#### 4.1.4 Use of non-study mRDTs

Although health workers were required to use SD Bioline *P*.*f* mRDTs during the study, survey and app usage results showed that health workers may have been under the impression that all mRDT brands used the same administrative steps. Even after the intervention, 43.9% of health workers noted in the endline survey that less than four diluent drops were required for mRDT administration, with 39.9% of health workers sharing that they used a non-study mRDT brand one or more times during the study. While the increase in the endline survey health worker recall of the number of diluent drops required was marginal, the in-app administration instructions which indicated the number of drops required may have aided health workers in using the right procedure while testing. While *HealthPulse* was designed to be compatible across multiple health areas and mRDT brands, this study was only conducted using SD Bioline *P*.*f*. The mRDT brands used in Kenya change on a yearly basis depending on the mRDTs procured by donors, such as the Global Fund [37], as well as brands purchased through the MOH. Future studies can aim to assess the feasibility and acceptability of the app when health workers are balancing the use of multiple available mRDT brands, which would more closely model a typical clinic environment.

#### 4.1.5 Readout and medication mismatch

Results showed mismatches between mRDT interpretation and ACT dispensing, with health workers dispensing ACTs to patients with negative mRDT readouts among 3.5% of encounters. Although one health worker was responsible for most of these encounters (68.2%), it is important to understand the reasons why a health worker would still provide an ACT when the mRDT result is negative and how to best mitigate these decisions moving forward. This study documented that these instances occurred when the patient had clear malaria symptoms, when the patient requested or demanded medication, when the health worker felt obligated to provide the patient with the medication, or when microscopy confirmed a positive result.

Additional studies have documented reasons why health workers have provided medication for a negative mRDT readout [38–41]. For example, a systematic review by Kabaghe et al. identified many factors influencing compliance to mRDT results, including years of work experience, patient expectations, trust in the result, and perceived accuracy of the result [38]. The review also found that community health workers (CHWs) had the highest compliance to negative test results compared to other health worker cadres, such as clinicians and nurses, indicating that health workers with more training are more likely to use clinical presentation and past patient experiences over the mRDT result as a standalone guide for malaria diagnosis. This trend was visible in our study, as more private clinic HWs than CHVs dispensed ACTs to a patient with a negative mRDT result. This highlights the need for increased technical training to improve health worker confidence that mRDTs are an effective and trustworthy diagnosis tool, which may be reinforced through continued *HealthPulse* use.

### 4.2 Acceptability

#### 4.2.1 *HealthPulse* user experience

Baseline and endline survey results showcased the app’s acceptability among health workers. While most health workers agreed that the app would be useful at baseline, this percentage increased after the intervention was introduced, with 99.5% of CHVs and 100% of private clinic HWs agreeing that the app was useful. Compared to the baseline responses, there was a large increase in agreement from both CHVs and private clinic HWs (~ 20% increase in each group) that the app’s pictures improved their skills for mRDT administration and interpretation. Additionally, after using the app, a higher percentage of health workers from both groups shared in the endline survey that the timer feature was helpful, that the capture of treatment information was helpful, and that they were confident that their results were saved.

Several studies have demonstrated the acceptability of mHealth interventions among different users. For example, a pilot study in Tanzania assessing the acceptability of an mHealth platform to facilitate the prevention of mother-to-child transmission of HIV found that health workers in hospitals and health centers had a significant improvement in their overall, average acceptability of the mHealth intervention by the end of the study [42]. Additionally, a study by Otieno et al. assessed the feasibility, use, and acceptability of a mobile phone text-messaging intervention to improve caregivers’ adherence to malaria care and post-review treatment for children with uncomplicated malaria in western Kenya. Their results showed that caregivers had a high-willingness to use the intervention, as all health workers reported that they would like to receive text-message reminders about giving their child medication, and 99.7% shared that they would like to receive a reminder to bring their child back for a post-treatment review [43]. As the willingness and acceptability of health workers to use mHealth interventions is a key factor to its sustained uptake, additional versions of *HealthPulse* will continue to assess user buy-in, especially as more advanced features are added.

#### 4.2.2 CHEW perspectives

When CHEWs were asked to respond to the endline survey regarding the app’s features, only 60.0% of CHEWs agreed that *HealthPulse* was easy for CHVs to use. This difference between the CHV and CHEWs responses is noteworthy, as CHEWs did not use the app themselves through their supervisor role and only relied on their own perceptions of their CHVs’ experiences. Nonetheless, almost 100% of both CHVs and private clinic HWs agreed that it would be helpful for their supervisor to have access to their work, showcasing the app as a potential supportive supervision tool which would be accepted by health workers. mHealth interventions for CHW supervision have been used elsewhere [44–46], although the evidence of their effectiveness remains limited.

#### 4.2.3 Integrating *HealthPulse* into existing workload

Compared to baseline survey results, fewer health workers indicated that they preferred the app to paper instructions or tracking after the intervention was introduced, although the difference in responses was minimal. While this could be due to new shifts in health worker workflows, it is important to understand which features in the app provide an extra benefit to health workers and which may lead to an unnecessary increase in their workload. Many studies have highlighted the need for health worker efficiency [47–49] with an enabling environment key to increasing health worker productivity. It is possible that the study requirement of needing to enter data into both *HealthPulse* and MOH paper forms may have biased the perspective of app efficiency for some. Future activities can assess the use of *HealthPulse* as a primary documentation and reporting tool, which could reduce workload and improve health worker efficiency by removing time spent on paper reporting.

### 4.3 Limitations

The study had several limitations. To begin, this pilot study only captured the utility and user experience of *HealthPulse* in one county in Kenya; therefore, it is possible that the app’s feasibility and acceptance may be different based on regional and country-level variance. For the sample sizes and health worker characteristics, there were many more health workers from the CHV cohort than the private clinic HW cohort. Future studies could aim to include a larger sample of private clinic HWs, who would provide a more holistic representation of the app perceptions in the private sector.

Health worker attrition was also a key limitation, especially as health workers dropped out due to issues with getting access to or maintaining access to a reliable smartphone. CHV attrition during the study was caused by three main factors 1) CHVs had smartphones that were not compatible with running the app, 2) CHVs claimed to have a personal smartphone at the beginning of the study, but it ended up being a shared phone with inconsistent access, and 3) CHVs lost their phone or it broke down. During study enrollment, health workers were only asked if they had a smartphone (Yes/No) but were not asked to provide details on phone ownership, such as its reliability or if the phone was shared. If the intervention was scaled up, additional resources would be needed to provide reliable access for all health workers to a smartphone while testing patients and app data uploads should be available free of charge to health workers. For the private clinic HW cohort, attrition was due to health workers leaving their job during the study period.

The survey tools also had a few limitations. For example, questions on the baseline and endline survey gauging potential support and the app experience were worded in support of the app; therefore, it is possible that this influenced health workers to more easily agree with these statements. Furthermore, this study did not gauge baseline viewpoints from the CHEWs, which limits the utility of their endline responses.

Additionally, the quality of data collected through the app was hindered by the upload of photos with previously photographed cassettes, which accounted for 16.7% of all mRDTs administered. The reasons for this test reuse behavior are being further explored in the follow-up qualitative study. AI enhancements are also being researched to identify mRDTs which are photographed more than once, outside of the manufacturer specified read window, or misadministered to further support supervision activities at scale.

The COVID-19 pandemic resulted in unforeseen challenges at the beginning of the study, which delayed the official start date and caused shortages in study supplies. Supply chain disruptions led to inconsistent availability of ACTs at participating clinics. To address these supply challenges, the National Malaria Control Program (NMCP) organized a redistribution of medications from clinics with a high supply to clinics with a low supply. Additionally, lockdowns and restrictions on group meeting sizes resulted in delayed health worker training. Other factors influencing the implementation timeline included a health worker strike (January 6th—February 24th, 2021) and a one-month mass bed net distribution campaign involving CHVs. After shifting study timelines to adjust for the pandemic, training moved forward and was conducted with all necessary staff and health workers. At the time of the study, COVID-19 RDTs were not yet available for use. If the same study were performed today during a COVID-19 outbreak, CHVs and private clinic HWs could be provided with both mRDTs and COVID-19 RDTs, which could improve the immediate guidance for health care staff and patients regarding the underlying cause of illnesses with similar symptoms.

### 4.4 Recommendations and next steps

Apart from its primary use by health workers as a testing aid, the many data points *HealthPulse* captures may allow for further benefits to the malaria community, including its potential as a quality improvement tool, through supportive supervision, mRDT interpretation, and/or surveillance actions. Auditing and surveillance practices could be enabled through mRDT result digitization to provide opportunities for supervisors to offer timely and targeted guidance to health workers. When combined with supervisor access to digitized mRDT images and AI algorithm mRDT interpretations, which could alert supervisors to cases where the health worker’s interpretation and the AI interpretation diverge, supervisors could support health workers more efficiently and promptly. Additionally, AI algorithms trained to interpret mRDTs can provide a consistent way to identify health worker challenges, such as a failure to identify faint lines on mRDTs.

With access to timely data, *HealthPulse* has potential as a malaria surveillance tool, enabling identification of malaria hotspots to further aid resource allocation and decision-making, particularly in settings that have not previously been captured in health management information systems. The mobile tools Solution for Community Health-workers (SOCH) and FeverTracker are two apps recently developed in India with malaria surveillance capabilities [50,51]. Both platforms have had early success in reducing the need for paper-based tools, assisting with supply chain management, and providing timely data on malaria incidence.

Future work, including follow-up studies and app updates, can provide additional evidence to explore the app’s utility. To begin, it is necessary to understand the facilitators and barriers in engaging with the app as well as reasons behind ongoing user errors among health workers. To explore this topic, the study team organized a qualitative study among health workers and CHEWs to gain insights on these issues, which will be presented elsewhere. Additionally, the team will separately review mRDT interpretations from the Panel Readers, AI algorithm, and PCR testing results. These results will allow the research team to measure how *HealthPulse* AI performs as an interpretation aid.

## 5. Conclusion

*HealthPulse* was designed as a supportive tool for mRDT testing, and these results highlight its potential for integrating the tool into health workers’ everyday use. mRDT implementation knowledge among health workers generally improved, but was not perfected post-intervention, and results indicated areas for app improvement. *HealthPulse* users in the study indicated strong support for future use and scale-up of this intervention, with support from both the community level and the private sector. Overall, these results demonstrate that an app to support health workers in low- and middle-income countries such as Kenya is a workable, acceptable, and feasible model with potential for scale up as a supportive supervision, interpretation, and/or surveillance tool.

## Supporting information

S1 Table Participant group definitions, inclusion, and exclusion criteria. Definitions and inclusion/ exclusion criteria provided for CHVs, private clinic HWs, CHEWs, and patients.
(PDF)

S2 Table Demographics of CHVs and private clinic HWs. Demographics include subcounty, facility type, facility ownership, region, gender, age at enrollment, highest educational attainment, years of health worker experience, years or malaria experience, preferred reading and speaking languages, and visual acuity.
(PDF)

S3 Table Demographics of CHEWs. CHEW demographics include subcounty, gender, age at enrollment, highest educational attainment, years of CHEW experience, and years of malaria experience.
(PDF)

S1 Fig Key highlights from the *HealthPulse* app. Important features of the app include instructional screens, an mRDT timer, interpretation guidance, and next steps for treatment. Reprinted from Audere under a CC BY license, with permission from Audere, original copyright Audere 2022.
(TIF)

S2 Fig Sample mRDT images provided in the endline survey. Health workers were asked to examine sample mRDT photos and determine if they were positive, negative, or invalid. Results were compared to responses for a similar exercise in the baseline survey, in order to assess changes in malaria mRDT implementation knowledge after using the intervention. Reprinted from Audere under a CC BY license, with permission from Audere, original copyright Audere 2022.
(TIF)

S1 File Endline survey. This survey was completed by both health worker groups (CHVs and private clinic HWs) after using *HealthPulse* to gauge acceptability and feasibility of app implementation.
(PDF)

S2 File Inclusivity in global research form. This form describes the ethical considerations, permits, and authorship of this study.
(PDF)

S3 File Full baseline and endline survey results. This data set contains the survey responses from CHVs and private clinic health workers, with surveys completed before and after they used the *HealthPulse* app.
(XLSX)

S4 File Process data on app use. This data set contains all data collected directly from the app, with results presented in sections 3.3 and 3.4.
(XLSX)
