# Supplementary material for: Use of a health worker-targeted smartphone app to support quality malaria RDT implementation in Busia County, Kenya: A feasibility and acceptability study
Source: PLoS One. 2024 Mar 26;19(3):e0295049. doi: 10.1371/journal.pone.0295049 (PMC10965099; doi:10.1371/journal.pone.0295049)
Supplement: S1 Table — Definitions and inclusion/ exclusion criteria provided for CHVs, private clinic HWs, CHEWs, and patients. (PDF) [file pone.0295049.s001.pdf]

Participant Group	Definition	Inclusion Criteria	Exclusion Criteria
**Community Health Volunteer (CHV)**	• Volunteers who provide support to individuals in their community for a variety of health promotion services, including malaria testing using mRDTs.	• CHVs who care for patients who may contract malaria.<br>• CHVs who have received standard training on malaria and the use of mRDTs.<br>• CHVs who have access to mRDTs and malaria treatment medication.<br>• CHVs who have access to a smartphone.	• CHVs who do not report to a CHEW.<br>• CHVs who live in sub-counties where another app is in use for managing CCM practices.<br>• Persons unable or unwilling to consent to the study.
**Private clinic health workers (private clinic HWs)**	• Health workers in the private sector including clinical officers, nurses, and lab technicians.	• PCHWs who are responsible for both patient diagnostic tests for malaria and dispensing anti-malarial medications.<br>• PCHWs who have received standard training on malaria and the use of RDTs.<br>• PCHWs who have access to mRDTs and malaria treatments.<br>• PCHWs who have access to a smartphone.	• PCHWs who are not responsible for both malaria diagnostic testing and dispensing anti-malarial medications.<br>• Persons unable or unwilling to consent to the study.
**Community Health Extension Worker**<br>**(CHEW)**	• Public sector health workers responsible for supervising community health volunteers. A group of roughly 10 CHVs typically report to one CHEW.	• CHEWs who manage CHVs providing mRDT tests and malaria treatment.	• Persons unable or unwilling to consent to the study.

**Patients**	• Individuals residing in Busia County who receive testing and care for potential malaria diagnosis from CHVs or PCHWs.	• Individuals for whom there is clinical suspicion of uncomplicated malaria based on fever and/or history of fever within the previous 48 hours.<br>• Male and female community members aged 18 years and above who are visited by CHVs in their respective households; including those who may be or are pregnant.<br>• Male and female community members between 10 years and 18 years who are visited by CHVs and whose parents/guardians provide consent and who provide assent themselves.<br>• Male and female outpatients age 18 years and above; including those who may be or are pregnant.	• Male and female outpatients (age 18 years and above and below 18 years) presenting with suspected complicated malaria. These individuals, if presented, were referred for immediate health care.<br>• Persons unable or unwilling to consent to the study.<br>• Male and female community members under 10 years of age.
