# Supplementary material for: Use of a health worker-targeted smartphone app to support quality malaria RDT implementation in Busia County, Kenya: A feasibility and acceptability study
Source: PLoS One. 2024 Mar 26;19(3):e0295049. doi: 10.1371/journal.pone.0295049 (PMC10965099; doi:10.1371/journal.pone.0295049)
Supplement: S2 Table — Demographics include subcounty, facility type, facility ownership, region, gender, age at enrollment, highest educational attainment, years of health worker experience, years or malaria experience, preferred reading and speaking languages, and visual acuity. (PDF) [file pone.0295049.s002.pdf]

Characteristics	Health Worker Demographics		
	Total (N=223)	CHVs (N=200)	Private clinic HWs (Nurses, Clinical Officers, Lab Technicians) (N=23)
	Frequency (%)	Frequency (%)	Frequency (%)
**Subcounty**			
Matayos	45 (20.2)	39 (19.5)	6 (26.1)
Nambale	17 (7.6)	14 (17.9)	3 (13.0)
Samia	49 (22.0)	45 (22.5)	4 (17.4)
Teso North	31 (13.9)	24 (12.0)	7 (30.4)
Teso South	81 (36.3)	78 (39.0)	3 (13.0)
**Facility Type**			
Clinic	16 (7.2)	0 (0.0)	16 (69.6)
Dispensary	134 (60.1)	134 (67.0)	0 (0.0)
Health Center	59 (26.5)	56 (28.0)	3 (13.0)
Hospital	6 (2.7)	4 (2.0)	2 (8.7)
Sub-district Hospital	1 (0.4)	0 (0.0)	0 (0.0)
Nursing Home	1 (0.4)	0 (0.0)	1 (4.3)
Other	6 (2.7)	6 (3.0)	1 (4.3)
**Facility Ownership**			
Faith Based/ Mission/ Church	9 (4.0)	6 (3.0)	3 (13.0)
Government/Council	192 (86.1)	192 (96.0)	0 (0.0)
NGO	4 (1.8)	1 (0.5)	3 (13.0)
Private	18 (8.1)	1 (0.5)	17 (73.9)
**Region**			
Peri Urban	27 (12.1)	21 (10.5)	6 (26.1)
Rural	174 (78.0)	167 (83.5)	7 (30.4)
Urban	22 (9.9)	12 (6.0)	10 (43.5)
**Gender**			
Female	149 (66.8)	137 (68.5)	12 (52.2)
Male	71 (31.8)	60 (30.0)	11 (47.8)
Other	3 (1.3)	3 (1.5)	0 (0.0)
**Age at enrollment**			
25-29 years	13 (5.8)	4 (2.0)	9 (39.1)
30-39 years	42 (18.8)	36 (18.0)	6 (26.1)
40-59 years	149 (66.8)	146 (73.0)	3 (13.0)

60+ years	19 (8.5)	14 (7.0)	5 (21.7)
**Highest Educational Attainment**			
Primary	69 (30.9)	69 (34.5)	0 (0.0)
Secondary	119 (53.4)	119 (59.5)	0 (0.0)
College (2 year degree)	34 (15.2)	12 (6.0)	22 (95.7)
University (4 year degree)	1 (0.4)	0 (0.0)	1 (4.3)
**Years of Health Worker Experience**			
0-4 years	26 (11.7)	13 (6.5)	13 (56.5)
5-9 years	51 (22.9)	50 (25.0)	1 (4.3)
10-14 years	57 (25.6)	57 (28.5)	0 (0.0)
15-19 years	65 (29.1)	64 (32.0)	1 (4.3)
20+ years	24 (10.8)	16 (8.0)	8 (34.8)
**Years of Malaria Experience**			
0-4 years	125 (56.1)	111 (55.5)	14 (60.9)
5-9 years	79 (35.4)	77 (38.5)	2 (8.7)
10-14 years	12 (5.4)	10 (5.0)	2 (8.7)
15-19 years	3 (1.3)	2 (1.0)	1 (4.3)
20+ years	4 (1.8)	0 (0.0)	4 (17.4)
**Preferred Reading Language**			
English	174 (78.0)	154 (77.0)	20 (87.0)
Kiswahili	49 (22.0)	46 (23.0)	3 (13.0)
**Preferred Speaking Language**			
English	32 (14.3)	21 (10.5)	11 (47.8)
Kiswahili	184 (82.5)	172 (86.0)	12 (52.2)
Other	7 (3.1)	7 (3.5)	0 (0.0)
**Reading/ Near Visual Acuity**			
Excellent vision (J1-J2)	12 (5.4)	8 (4.0)	4 (17.4)
Average vision (J3-J8)	139 (62.3)	122 (61.0)	17 (73.9)
Poor vision (J9-J11)	72 (32.3)	70 (35.0)	2 (8.7)
