# Supplementary material for: Use of a health worker-targeted smartphone app to support quality malaria RDT implementation in Busia County, Kenya: A feasibility and acceptability study
Source: PLoS One. 2024 Mar 26;19(3):e0295049. doi: 10.1371/journal.pone.0295049 (PMC10965099; doi:10.1371/journal.pone.0295049)
Supplement: S3 Table — CHEW demographics include subcounty, gender, age at enrollment, highest educational attainment, years of CHEW experience, and years of malaria experience. (PDF) [file pone.0295049.s003.pdf]

CHEW Demographics (N=45)	
**Characteristics**	**Frequency (%)**
**Subcounty**	
Matayos	11 (24.4)
Nambale	5 (11.1)
Samia	9 (20.0)
Teso North	3 (6.7)
Teso South	17 (37.8)
**Gender**	
Female	30 (66.7)
Male	15 (33.3)
**Age at enrollment**	
25-29 years	14 (31.1)
30-39 years	22 (48.9)
40-59 years	7 (15.6)
60+ years	2 (4.4)
**Highest Educational Attainment**	
College	30 (66.7)
Secondary	1 (2.2)
University	14 (31.1)
**Years of CHEW Experience**	
Less than 1 year	3 (6.7)
1-2 years	17 (37.8)
3-4 years	11 (24.4)
5-6 years	3 (6.7)
6+ years	11 (24.4)
**Years of Malaria Experience**	
1-2 years	24 (53.3)
3-4 years	11 (24.4)
5-6 years	5 (11.1)
6+ years	5 (11.1)
