# Supplementary figures and images for: Use of a health worker-targeted smartphone app to support quality malaria RDT implementation in Busia County, Kenya: A feasibility and acceptability study
Source: PLoS One. 2024 Mar 26;19(3):e0295049. doi: 10.1371/journal.pone.0295049 (PMC10965099; doi:10.1371/journal.pone.0295049)

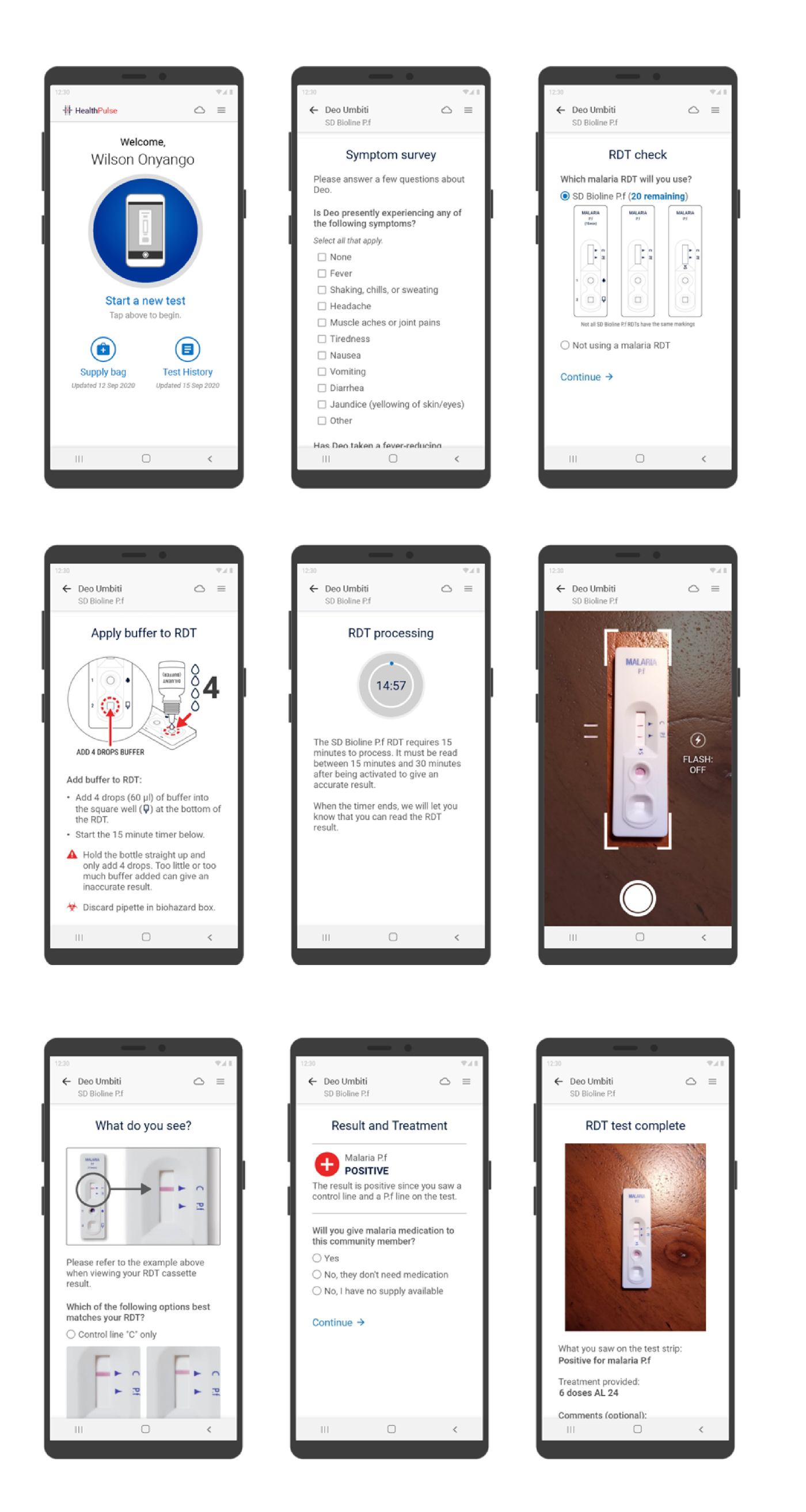

Supplement: S1 Fig — Important features of the app include instructional screens, an mRDT timer, interpretation guidance, and next steps for treatment. Reprinted from Audere under a CC BY license, with permission from Audere, original copyright Audere 2022. (TIF) [file pone.0295049.s004.tif]

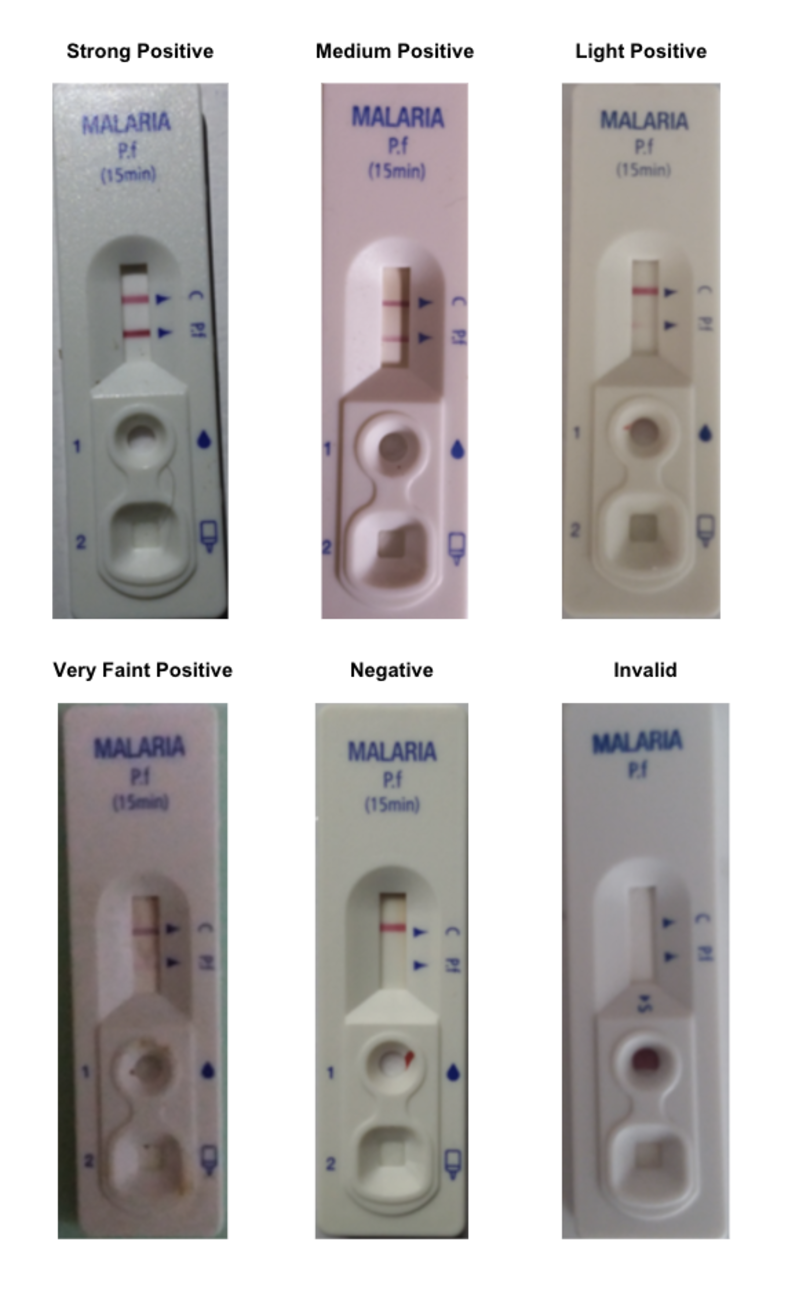

Supplement: S2 Fig — Health workers were asked to examine sample mRDT photos and determine if they were positive, negative, or invalid. Results were compared to responses for a similar exercise in the baseline survey, in order to assess changes in malaria mRDT implementation knowledge after using the intervention. Reprinted from Audere under a CC BY license, with permission from Audere, original copyright Audere 2022. (TIF) [file pone.0295049.s005.tif]
