# Supplementary material for: Use of a health worker-targeted smartphone app to support quality malaria RDT implementation in Busia County, Kenya: A feasibility and acceptability study
Source: PLoS One. 2024 Mar 26;19(3):e0295049. doi: 10.1371/journal.pone.0295049 (PMC10965099; doi:10.1371/journal.pone.0295049)
Supplement: S1 File — This survey was completed by both health worker groups (CHVs and private clinic HWs) after using HealthPulse to gauge acceptability and feasibility of app implementation. (PDF) [file pone.0295049.s006.pdf]

Final frontline health worker survey

Presented at the end of the study period.

**CLIENT INFORMATION SHEET**
PLEASE READ TO THE RESPONDENT:
Hello, my name is ________ and I am conducting surveys today on behalf of Population Services Kenya (PSK).<br>Thank you for participating in the mRDT study in Busia County. In this survey, we are interested in learning more about your experiences using the OpenRDT app during the study.<br>The questions will take about 20 minutes to complete. All information that you provide us will remain strictly private and confidential. We will not discuss your individual answers with the staff members.<br>If you have questions about this survey, please ask the person administering the survey or contact xxx on xxx or xxx.<br>Do you have any questions at this time?
I certify that I have read the Client Information Sheet and have explained this survey to the participant, and that they have previously consented to participate in the study. They have been given the opportunity to ask questions, which have been answered satisfactorily.<br><br>**Name of interviewer:** ____ **Position:** ________________________________<br><br>**Signature:** ____ **Date of interview:** _______________________

**IDENTIFICATION**

County: Busia

Sub-county: [___] Bunyala [___] Butula [___] Matayos [___] Nambale [___] Samia [___] Teso North [___] Teso South

Ward name: [____________________________________]

Link / Facility name: [____]

Community Unit name: [____]

Location: [___] Urban [___] Peri-urban [___] Rural

Health Worker cadre:

[___] Clinical Officer [___] Nurse [___] Lab Technician [___] CHEW [___] CHV [___] Other Specify________________________

CHV ID:____ Name of Health Worker: ___________________________________

Name of Supervisor / CHEW: ___________________________________

	SUPERVISOR	EDITED BY	ENTERED BY
NAME	______________________	______________________	______________________
DATE	______________________	______________________	______________________

Please answer the following questions to help us understand your perceptions of the OpenRDT app. All questions are required unless indicated.

NO.	QUESTION	RESPONSE OPTIONS	CODES	SKIP
Q101	What is your overall impression of the malaria OpenRDT application?	Not useful	0	
		A little useful	1	
		Fairly useful	2	
		Very useful	3	
Q102	Using the malaria OpenRDT app was:	Very difficult	1	
		Somewhat difficult	2	
		Easy	3	
		Very easy	4	
Q103	I trusted the information shown to me in the OpenRDT app.	Strongly disagree	1	
		Somewhat disagree	2	
		Somewhat agree	3	
		Strongly agree	4	
Q104	The instructions in the OpenRDT app were clear and helpful.	Strongly disagree	1	
		Somewhat disagree	2	
		Somewhat agree	3	
		Strongly agree	4	
Q105	I prefer using the OpenRDT app to using paper instructions and paper tracking sheets.	Strongly disagree	1	
		Somewhat disagree	2	

		Somewhat agree	3	
		Strongly agree	4	
**Q106**	Using the OpenRDT app vs. using paper instructions and paper tracking sheets could save me time when working with community members.	Strongly disagree	1	
		Somewhat disagree	2	
		Somewhat agree	3	
		Strongly agree	4	
**Q107**	The timers in the OpenRDT app helped me understand when the RDT result was ready to be viewed.	Strongly disagree	1	
		Somewhat disagree	2	
		Somewhat agree	3	
		Strongly agree	4	
**Q108**	I found it helpful to see sample images of the mRDT in the app when reading the result.	Strongly disagree	1	
		Somewhat disagree	2	
		Somewhat agree	3	
		Strongly agree	4	
**Q109**	I felt confident when I indicated whether the RDT is positive, negative, or unreadable.	Strongly disagree	1	
		Somewhat disagree	2	
		Somewhat agree	3	
		Strongly agree	4	
**Q110**	I found it helpful that the OpenRDT app captured treatment information (such as the medications I gave to community members).	Strongly disagree	1	
		Somewhat disagree	2	
		Somewhat agree	3	
		Strongly agree	4	
**Q111**	I found it useful that my supervisor was able to access my RDT results and malaria treatment data on a regular basis, versus waiting for me to send in my paper form.	Strongly disagree	1	
		Somewhat disagree	2	
		Somewhat agree	3	
		Strongly agree	4	
**Q112**	I found it easy to use OpenRDT's tracking of how many RDTs and malaria treatments I had remaining in my bag.	Strongly disagree	1	
		Somewhat disagree	2	
		Somewhat agree	3	
		Strongly agree	4	
**Q113**	If OpenRDT provided the RDT interpretation directly in the app (instead of me needing to indicate whether it was positive or negative), I would likely trust that result.	Strongly disagree	1	
		Somewhat disagree	2	
		Somewhat agree	3	
		Strongly agree	4	
**Q114**	It would be useful to have an app to provide more information about malaria testing happening in my area and surrounding areas.	Strongly disagree	1	
		Somewhat disagree	2	
		Somewhat agree	3	
		Strongly agree	4	
**Q115**	I believe having an image saved of the RDT is useful for the following reasons:<br><br>***(select all that apply)***	The image proves I did an RDT test for the community member.	1	
		The picture can sometimes help me see if the test result was positive or negative.	2	
		I am confident that my RDT results are being properly saved.	3	
		I like that my supervisor can more quickly see the efforts of my work.	4	
		Other reason (specify) ________________	88	

		I do not believe having an image of the RDT saved is useful	0	
Q116	Did you often find it difficult to get a good photo of the RDT?	Yes	1	
		No	0	
Q117	When you needed to retake a photo of the RDT, what were the main reasons?<br><br>Select all that apply.	Too dark	1	
		Too light	2	
		Too blurry	3	
		Too big	4	
		Too small	5	
		Too many shadows	6	
		RDT not aligned	7	
		Other, Specify	__________	
Q118	When you took photos of RDTs, what type of light was typically present?	Inside using sunlight from a window	1	
		Inside using a lamp or other light	2	
		Outside using sunlight<br>Other, Specify	3<br>_________	
Q119	Did you have challenges using a mobile phone while testing individuals?<br><br>Select all that apply.	No challenges	1	
		Hard to keep phone clean	2	
		Hard to keep phone charged	3	
		Hard to handle both the phone and test supplies	4	
		Hard to find a surface to put phone on	5	
		Other, specify	________	
Q120	The text size in the app is:	Too big	1	
		Just right	2	
		A bit too small	3	
		A lot too small	4	
Q121a	Is there anything else you would have found useful in the OpenRDT app?	Yes	1	
		No	0	**Skip to Q122**
Q121b	What would you have found useful in the OpenRDT app?	Specify	________	
		Don't know	98	
Q122	Since the beginning of the study, have you started wearing spectacles or contact lenses?	Yes	1	
		No	2	
		I plan to get spectacles/contact	3	
Q123	Please provide any additional comments you have regarding the OpenRDT app or your participation in the study.	Specify ________________		
**SECTION 4: mRDT PRACTICES**				
Q124	For the SD Bioline Pf mRDT, how many drops of diluent do you add?	1	1	
		2	2	
		3	3	
		4	4	
		5	5	
		6	6	
		7	7	

**Q125**	For the SD Bioline Pf mRDT, how long do you wait until reading the test result?	5 min	1	
		10 min	2	
		15 min	3	
		20 min	4	
		25 min	5	
**Q125**	If you read the mRDT after this time period you may get a false result.	10 min	1	
		20 min	2	
		30 min	3	
		40 min	4	
		50 min	5	
**Q127**	What result lines do you consider positive on the mRDT? **(select all that apply)**	Strong visible lines	1	
		Medium visible lines	2	
		Faint visible lines	3	
		Very faint visible lines	4	
**Q128**	If the control line doesn't show on the test, but a Pf line does show, what does that mean?	The test result is negative	1	
		The test result is positive	2	
		There is a problem with the test, I should use another test kit	3	
**Q129**	For each of the following test images, what result is it showing (positive, negative, or invalid)? (**See sample images in S2 Fig**)	Image 1: ______________		
		Image 2: ______________		
		Image 3: ______________		
		Image 4: ______________		
		Image 5: ______________		
		Image 6: ______________		
		Image 7: ______________		

(See last question on the next page)

SECTION 5: TRAINING AND SUPERVISION RELATED TO mRDT<br>Next, we are going to ask you about any training and supervision related to mRDT.				
NO.	QUESTION	RESPONSE OPTIONS	CODES	SKIP
Q130a	Have you attended malaria RDT training?	Yes	1	
		No	0	Go to Q126a
Q130b	Date of last training?<br>(Enter Month-year)	[___|___]-[___|___]		
Q131a	Did you have any supervisory visits regarding malaria case management in the last 1 month?	Yes	1	
		No	0	**End**
Q131b	Was malaria case management/community case management in malaria (CCMm) a topic of any of these visits?	Yes	1	
		No	0	**End**
Q131c	What did these visits include related to malaria case management?<br>(Select all that apply)	Review of the mRDT testing or sample collection procedure	1	
		Discussions on treating uncomplicated malaria	2	
		Provision of feedback	3	
		Record keeping	4	

Thank you for your participation in our study and your time filling out this survey.

INTERVIEWER'S COMMENTS

____
