# Supplementary material for: Use of a health worker-targeted smartphone app to support quality malaria RDT implementation in Busia County, Kenya: A feasibility and acceptability study
Source: PLoS One. 2024 Mar 26;19(3):e0295049. doi: 10.1371/journal.pone.0295049 (PMC10965099; doi:10.1371/journal.pone.0295049)
Supplement: S2 File — This form describes the ethical considerations, permits, and authorship of this study. (PDF) [file pone.0295049.s007.pdf]

Inclusivity in global research

PLOS' policy on inclusivity in global research aims to improve transparency in the reporting of research performed outside of researchers' own country or community and ensures that PLOS publications reporting global research adhere to high standards for research ethics and authorship. Authors of relevant research articles may be asked to complete the questionnaire below, which outlines ethical, cultural, and scientific considerations specific to inclusivity in global research. This questionnaire may be requested when researchers have travelled to a different country to conduct research, if research uses samples collected in another country, research with Indigenous populations or their lands, or if research is on cultural artefacts. Researchers travelling to another country solely to use laboratory equipment will not normally be required to complete the questionnaire. However, the questionnaire can be requested at the journal's discretion for any submission – if you have been requested to complete this questionnaire by the PLOS journal you submitted to, please do so.

Please complete the questionnaire below and include this as a Supporting Information file with your manuscript. Note that if your paper is accepted for publication, this checklist will be published with your article in the supporting information files. Please ensure that you reference the checklist in the main body of your manuscript. We suggest adding a subsection 'Inclusivity in global research' to your Methods section and adding the following sentence: "Additional information regarding the ethical, cultural, and scientific considerations specific to inclusivity in global research is included in the Supporting Information (SX Checklist)"

The questions have been designed to be applicable to a wide range of study types, and there are subsections for both human subjects research and non-human subjects research. If any of the questions are not relevant to your research please mark them as "N/A" as appropriate.

Ethical considerations, permits and authorship

This section is applicable to all research types.

Provide details as to who granted permissions and/or consent for the study to take place in the Methods section of your manuscript. This should include the names of **all** ethics boards, governmental organizations, community leaders or other bodies that provided approval for the study. If individuals provided approval refer to these people by their role or title but do not list their name(s).

After initial IRB approval, in May 2021 the health worker survey tool was amended and approved by the IRB to add context on the user experience of smartphones and the application. In Jan 2022, the study was amended and approved by the IRB to add in-depth interviews (IDIs) with a set of frontline workers and CHEWS who participated in the study to gain a deeper understanding of quantitative responses from the surveys, as well as from key observations during the intervention. Institutional review board (IRB) approval was received from AMREF Health Africa Kenya before the study began (Ref: AMREF-ESRC P897/2020). PS Kenya conducted study activities under a research license from the National Commission for Science, Technology & Innovation, Republic of Kenya. The license covered malaria research in Busia county. License no: (NACOSTI/P/20/6252).The Busia County Director of Health was the signatory on a certificate of training that was provided to each of the frontline health workers who participated in the program. The health workers surveys were jointly designed by the study team and the Kenya MOH, and usability tested with a random sample of health workers before deployment.

If there were any deviations from the study protocol after approval was obtained please provide details of these changes in the Methods section of your manuscript.
Did this study involve local collaborators that are residents of the country where the research was conducted or members of the community studied? If you do not have any authors from said communities, please provide an explanation for this below.

Everyone listed as an author should meet PLOS' criteria for authorship and all individuals who meet these

Yes, the PS Kenya team and the PSI Digital Health and Monitoring Team, who are also included as authors on this paper, are Kenyan residents who fully participated and led this research project in Kenya; including training, data collection, and analysis.

criteria should be included in the author byline, rather than the acknowledgements. For further information please see the journal's Authorship Policy.

Human subjects research (e.g. health research, medical research, cross-cultural psychology)

Did you obtain written informed consent from a representative of the local community or region before the research took place? How did you establish who speaks for the community? Details of written informed consent obtained from study participants should be reported separately in the Methods section of your manuscript.

A project manager at Population Services Kenya organized conversations with Community Health Extension Workers (CHEWs) in sub-counties of Busia county where PS Kenya ran malaria case management programs and private clinic health facility managers in Busia county to invite them to participate in the study. As representatives of the local communities in which they worked, the CHEWs invited CHVs they supervised and private clinic health facility managers invited their clinic staff to participate in the study.

How did members of the local community provide input on the aims of the research investigation, its methodology, and its anticipated outcome(s)?

PS Kenya study leads validated the overall project goals with research officers from the Kenya Medical Research Institute (KEMRI). For example, the PSKenya team held a half-day 'Entry meeting' with the Busia County Health Department to discuss the study. A PS Kenya team member presented on the study and there was a discussion with the county team on issues related to their support in supervision of activities, data review and support to the lab.

When engaging with the local community, how did you ensure that the informed consent documents and other materials could be understood by local stakeholders?

All the informed consent documents were translated into the local language of Kiswahili.

Will the findings of the research be made available in an understandable format to stakeholders in the community where the study was conducted (e.g. via a presentation, summary report, copies of publications, etc.)? Please provide details of how this will be achieved.

Copies of publications will be shared with community stakeholders. Key findings were previously shared via presentations with the Kenya MoH.

Non-human subjects research using specimens/ animals collected as part of the study, or those housed in archival collections. Examples include archaeology, paleontology, botany and zoology.

Did the permission you obtained from a local authority to perform the study include an agreement on access to outputs and benefit sharing? This may include procedures to enable fair distribution of the benefits and resources arising from the research performed. Please include any details of Prior Informed Consent and Benefit Sharing Agreements obtained. These may be required by field-specific regulations, for example the Convention on Biological Diversity (CBD) and the associated Nagoya Protocol.

N/A

If the material used in your study was imported, please A) provide the year it was imported and B) indicate whether permits were obtained to import/export the materials used, C) provide details of any permits obtained. If this information is not available, please indicate this.

N/A

If you used archival specimens, please state how the material used in your study was acquired by the institute it is held in and provide details of any permits obtained for the original excavations/ sample collection. If this information is not available, please indicate this.

N/A

How was the potential cultural significance of the materials collected in your study to local communities considered in your research design? Were Indigenous peoples and/or local researchers and institutions involved with archaeological excavations / collection of specimens? If so, please provide a description of their involvement.

N/A

If your manuscript includes photographs of human remains please indicate whether authors obtained permission from descendants or affiliated cultural communities to do so.

N/A
